# Impeding Nucleotide‐Binding Oligomerization Domain‐Like Receptor 3 Inflammasome Ameliorates Cardiac Remodeling and Dysfunction in Obesity‐Associated Cardiomyopathy

**DOI:** 10.1161/JAHA.124.035234

**Published:** 2024-11-27

**Authors:** Shi‐qiang Liu, Sai‐yang Xie, Tong Zhang, Heng Zhang, Meng‐Ya Chen, Yun Xing, Nan Zhao, Lanlan Li, Si Chen, Sha‐sha Wang, Xiao‐feng Zeng, Wei Deng, Qi‐Zhu Tang

**Affiliations:** ^1^ Department of Cardiology Renmin Hospital of Wuhan University Wuhan PR China; ^2^ Hubei Key Laboratory of Metabolic and Chronic Diseases Wuhan PR China; ^3^ Cardiovascular Research Institute of Wuhan University Wuhan PR China

**Keywords:** heart failure, MCC950, mitochondrial reactive oxygen species, MitoTEMPO, nuclear factor kappa B, TXNIP, Heart Failure, Cardiomyopathy, Remodeling

## Abstract

**Background:**

Inflammation and metabolic disturbances are key culprits in the pathogenesis of obesity‐associated cardiomyopathy. The NLRP3 (nucleotide‐binding oligomerization domain‐like receptor 3) inflammasome mediates the release of the proinflammatory cytokines IL‐1β (interleukin‐1β) and IL‐18 by activating caspase‐1, which is strongly implicated in metabolic disturbances. We here sought to determine whether NLRP3 inflammasome inhibition could ameliorate obesity cardiomyopathy and if so, to further explore its underlying mechanisms.

**Methods and Results:**

Male mice were fed a high‐fat diet for 24 weeks to induce obesity cardiomyopathy. MCC950 was used to inhibit NLRP3 inflammasome activation. Recombinant adeno‐associated virus serotype 9 encoding TXNIP (thioredoxin‐interacting protein) under cTnT (cardiac troponin T) promoter and the mitochondrial‐targeted antioxidant MitoTEMPO were injected into obese mice to investigate the specific mechanism. To mimic obesity cardiomyopathy in vitro, neonatal rat ventricular myocytes transfected with the small interfering RNA against TXNIP were incubated with 400 μmol palmitic acid for 24 hours. NLRP3 inflammasome was significantly increased in obese hearts. NLRP3 inflammasome inhibition by NLRP3 deletion or MCC950 prevented obesity‐induced cardiac systolic and diastolic dysfunction, myocardial hypertrophy and fibrosis, and excessive lipid accumulation in male mice. Conversely, TXNIP overexpression worsened obesity‐associated cardiomyopathy. Similarly, MCC950 treatment or TXNIP knockdown reduced palmitic acid‐induced NLRP3 inflammasome activation and lipid storage. Mechanistically, abnormal NF‐κB (nuclear factor kappa B) pathway activation, increased mitochondrial reactive oxygen species, and elevated TXNIP levels led to excessive NLRP3 inflammasome activation.

**Conclusions:**

Our study confirms that aberrant NLRP3 inflammasome activation in cardiomyocytes worsens obesity‐associated cardiomyopathy and implicates inhibition of NLRP3 inflammasome as a potent therapeutic approach for obesity cardiomyopathy.

**Nonstandard Abbreviations and Acronyms**
AAV9: adeno‐associated virus serotype 9
ASC: apoptosis‐associated speck like protein
FAO: fatty acid oxidation
HFD: high‐fat diet
ND: normal diet
NF‐κB: nuclear factor kappa B
NLRP3: nucleotide‐binding oligomerization domain‐like receptor 3
NRVMs: neonatal rat ventricular myocytes
ROS: reactive oxygen species
TRX: thioredoxin
TXNIP: thioredoxin‐interacting protein

**Clinical Perspective**

**What Is New?**

- Our study reveals that mitochondrial reactive oxygen species‐mediated TXNIP/NLRP3 (thioredoxin‐interacting protein/nucleotide‐binding oligomerization domain‐like receptor 3) inflammasome activation in cardiomyocytes plays a critical role in the pathogenesis of obesity cardiomyopathy. Inhibition of NLRP3 inflammasome improves high‐fat diet‐induced cardiac dysfunction, lipid accumulation and myocardial remodeling.

**What Are the Clinical Implications?**

- Approaches to inhibit NLRP3 inflammasome activation might be achieved to mitigate obesity‐associated cardiomyopathy.
- The present study provided evidence that MCC950 and MitoTEMPO improved cardiac metabolic remodeling and further supported its clinical benefit in patients with obesity.

The prevalence of obesity has increased worldwide over the past 2 decades, making it a major health problem in the 21st century.^1^, ^2^ Patients with obesity are susceptible to a unique form of cardiovascular disease termed obesity cardiomyopathy, which develops independently of hypertension, coronary artery disease, and valvular heart diseases.^3^ Longstanding obesity is strongly associated with cardiac remodeling, leading to left ventricular hypertrophy and myocardial fibrosis.^4^ Early obesity cardiomyopathy is characterized by diastolic dysfunction, as evidenced by reduced myocardial compliance and elevated left ventricular filling pressure, whereas cardiac systolic function is either normal or mildly elevated.^5^ However, prolonged obesity impairs cardiac systolic function, as evidenced by reduced left ventricular shortening and a slight decrease in left ventricular ejection fraction.^6^ Adipose tissue dysfunction, systemic inflammation, metabolic disturbances, and mitochondrial damage are responsible for the pathogenesis of obesity cardiomyopathy.^3^ Accordingly, effective strategies to suppress inflammation and improve metabolic disorders may provide new therapeutic approaches for obesity cardiomyopathy.

NLRP3 (nucleotide‐binding oligomerization domain‐like receptor 3) inflammasome is a cytoplasmic supramolecular complex mediating caspase‐1 activation and release of proinflammatory factors IL‐1β (interleukin‐1β) and IL‐18.^7^ In response to endogenous danger signals, like microbial infection and environmental irritants, NLRP3 oligomerizes in the cytoplasm, recruits the adapter protein ASC (apoptosis‐associated speck like protein), and eventually forms a single fibrillar aggregate known as ASC speck.^8^ ASC speck provides a molecular platform for the recruitment of procaspase‐1, and caspase‐1 aggregated on ASC undergoes self‐cleavage to form p20 and p10 complexes with its protein hydrolyzing activity, which mediate mature IL‐1β and IL‐18 generation.^9^ The pro‐inflammatory cytokine IL‐1β contributes to myocardial hypertrophy and heart failure via cardiomyocyte hypertrophy induction and cardiac fibroblasts and immune cell activation.^10^ Previous studies have revealed that inhibiting NLRP3 inflammasome activation in macrophages and thereby attenuating myocardial IL‐1β and IL‐18 levels effectively ameliorates myocardial ischemic injury and pressure overload‐induced heart failure and cardiac remodeling.^11^, ^12^, ^13^ Recently, NLRP3 inflammasome activation in cardiomyocytes has received increasing attention because its inhibition significantly attenuates pressure overload‐induced cardiac hypertrophy, fibrosis, and cardiac dysfunction.^14^, ^15^ Elevated NLRP3 Inflammasome activity in cardiomyocytes leads to cardiomyocyte dysfunction and atrial fibrillation.^16^ However, whether NLRP3 inflammasome is activated in obesity cardiomyopathy and whether its activation contributes to the progression of heart failure remains poorly understood. Furthermore, whether the inhibition of NLRP3 inflammasome activation in cardiomyocytes improves obesity‐induced cardiac dysfunction remains unknown.

Mitochondrial dysfunction, including massive reactive oxygen species (ROS) production and impaired mitochondrial autophagy, is vital in the pathogenesis of obesity cardiomyopathy.^3^ In the setting of obesity, the energy supply of the heart is progressively supplied by fatty acid oxidation (FAO), along with increased lipid accumulation in the heart. Mitochondrial dysfunction leads to impaired FAO, thereby exacerbating cardiac lipotoxicity‐induced cardiac dysfunction.^17^ Imbalance of fatty acid transport, storage, and oxidation in cardiomyocytes leads to cardiac lipotoxicity. Collectively, reducing the fatty acid supply or improving FAO alleviates cardiac lipotoxicity and improves cardiac dysfunction in obesity cardiomyopathy. The TRX (thioredoxin) system is a critical intracellular antioxidant system that is negatively regulated by TXNIP (thioredoxin‐interacting protein) by binding to TRX and inhibiting its antioxidant activity.^18^ Upon ROS stimulation, TXNIP detaches from the TXNIP‐TRX complex and binds to NLRP3, promoting NLRP3 inflammasome activation and production of proinflammatory cytokines IL‐1β and IL‐18.^19^ However, whether TXNIP/NLRP3 inflammasome activation contributes to the development of obesity cardiomyopathy remains unknown.

In this study, we demonstrate that inhibition of NLRP3 inflammasome activity by NLRP3 deletion or injection of MCC950 prevented obesity‐induced cardiac dysfunction, pathological cardiac remodeling, and excessive lipid accumulation. Mechanistically, abnormal NF‐κB (nuclear factor kappa B) pathway activation, increased mitochondrial ROS production, and elevated TXNIP levels contribute to the excessive enhancement of NLRP3 inflammasome activity in heart of obese mice. These data imply that impeding NLRP3 inflammasome by MitoTEMPO or MCC950 represents a viable therapeutic strategy for obesity‐associated cardiomyopathy.

## METHODS

More detailed methods are described in Data S1.

### Data Availability

The data that support the findings of this study are available from the corresponding author upon request for purposes of reproducing the results or replicating the procedure.

### Animal Studies and Ethics

All animal experiments were performed following the *Guide for the Care and Use of Laboratory Animals* (National Institutes of Health Publication, revised 2011) and were approved by the Animal Care and Use Committee of the Wuhan University People's Hospital (IvD number: WDRM 20210704A). All mice were kept in a specific pathogen‐free environment at a suitable temperature (20–25 °C) and humidity (50±5%). The mice had free access to food and water. Mice were acclimatized for 1 week before the experiments. Mice were fed a high‐fat diet (HFD; Xietong Shengwu Research Institute, D12492, 60 kcal% fat) for 24 weeks to induce obesity cardiomyopathy as described previously.^17^, ^43^Control mice were fed a normal diet (ND) for 24 weeks (Table S1).

### Chemicals

Selective NLRP3 inhibitor MCC950 sodium (HY‐12815A) and mitochondria ROS scavenger MitoTEMPO (HY‐112879) were obtained from from Medchem Express (Shanghai, China). Palmitic acid (H8780) was purchased from Solarbio (Beijing, China). The GTVision+Detection System/Mo&Rb reagent purchased from Gene Technology (Shanghai, China). The Alexa Fluor 488‐ and 568‐goat antirabbit or mouse secondary antibodies were purchased from LI‐COR Biosciences (Lincoln, NE, USA). Primary antibodies against NLRP3, TXNIP, TRX, BNP (B‐type natriuretic peptide), and collagen I were purchased from Abcam (Cambridge, UK). Anti‐caspase‐1 antibody was acquired from Santa Cruz Biotechnology (Dallas, TX, USA). Antilamin B1 antibody was purchased from Proteintech (Rosemont, IL, USA). Primary antibodies against ASC, IL‐1β, and IL‐18 were acquired from ABclonal Technology (Wuhan, China). Primary antibodies for CTGF (connective tissue growth factor), NF‐κB, p‐NF‐κB (phosphorylated NF‐κB), and GAPDH were obtained from CST (Massachusetts, USA). All primary antibodies were shown in Table S2.

### Quantitative Real‐Time Polymerase Chain Reaction

To detect the expression of genes associated with cardiac hypertrophy, fibrosis, inflammation and fatty acid oxidation, total RNA was extracted from left ventricle tissue or neonatal rat ventricular myocytes (NRVMs) using TRIzol reagent (Invitrogen, 15596‐026) and reverse‐transcribed to cDNA by the Transcriptor First Strand cDNA Synthesis Kit (Roche Diagnostics, Switzerland). The cDNA fragments were amplified using the Roche LightCycler 480 detection system. The polymerase chain reaction (PCR) conditions were as follows. PCR conditions: 95 °C for 2 minutes, 95 °C for 10 seconds, 60 °C for 30 seconds, a total of 40 cycles. All primers were shown in Table S3. The relative mRNA levels were normalized to *GAPDH*.

### Statistical Analysis

All results are presented as the mean±SEM. All results were tested for normality with the Shapiro–Wilk normality test, and all data exhibited a normal distribution in the study. Two‐group comparisons were performed by unpaired Student's *t* test, and data were analyzed by 2‐way ANOVA and the Tukey posttest for comparison of multiple groups. Furthermore, one‐way ANOVA was used for the time‐course experiments. All statistical analyses were performed using GraphPad Prism 8.0 software.

## RESULTS

### NLRP3 Inflammasome Is Activated in Obesity Cardiomyopathy

**Figure 1:**
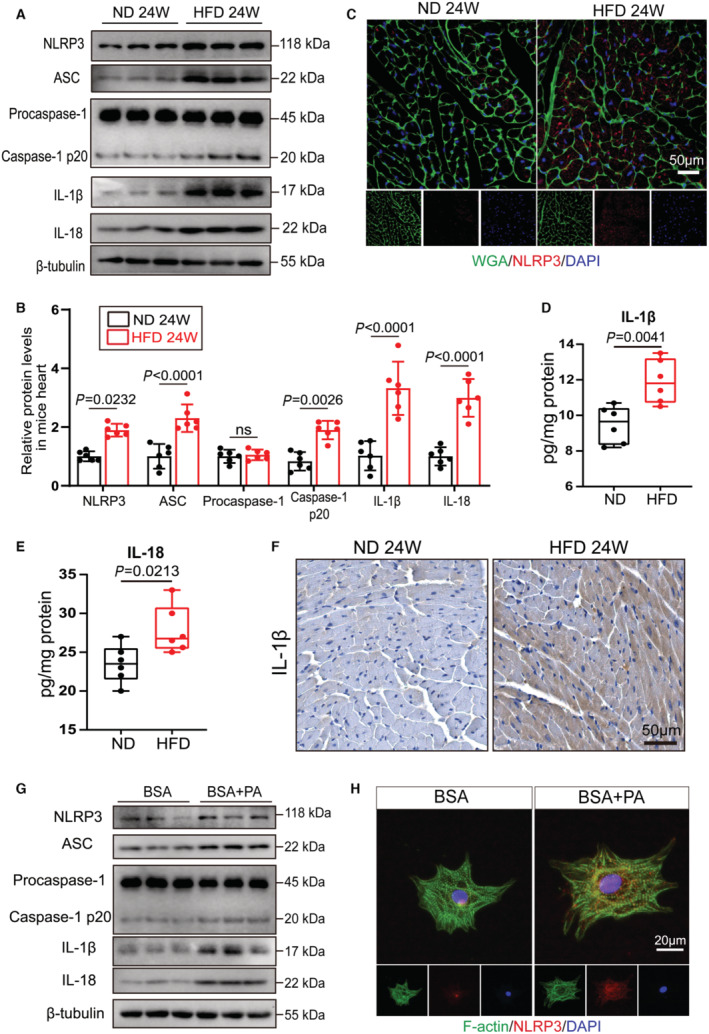
NLRP3 inflammasome is activated in obesity cardiomyopathy. **A**, **B**, Representative immunoblots (**A**) and quantification (**B**) of NLRP3, ASC, procapase‐1, cleaved caspase‐1 p20, IL‐1β, and IL‐18 in the heart of WT mice subjected to either ND or HFD consumption for 24 wks (n=6 per group). (**C**) Representative images of wheat germ agglutinin staining (green) and immunofluorescence staining of NLRP3 (red) in the heart of WT mice fed an HFD or ND for 24 wks. **D**, **E**, Protein of IL‐1β (**D**) and IL‐18 (**E**) in the heart of WT mice fed an HFD or ND for 24 wks. IL‐1β and IL‐18 protein were detected by ELISA. Values were normalized to total protein level (n=6 per group). **F**, Representative images of immunohistochemical staining of IL‐1β in the heart of WT mice subjected to either ND or HFD consumption for 24 wks. (**G**) Representative immunoblots of NLRP3, ASC, procapase‐1, cleaved caspase‐1 p20, IL‐1β, and IL‐18 protein in NRVMs treated with BSA or BSA+PA for 24 h. **H**, Representative images of immunofluorescence staining of NLRP3 (red) and F‐Actin (green) in NRVMs treated with BSA or BSA+PA. Significance was assessed by Student *t* test. Data are shown as the mean±SEM. ASC indicates apoptosis‐associated speck like protein; HFD, high‐fat diet; BSA, Bovine Serum Albumin; IL, interleukin; ND, normal diet; ns, not significant; NLRP3, nucleotide‐binding oligomerization domain‐like receptor 3; NRVM, neonatal rat ventricular myocyte; PA, palmitic acid; and WT, wild type.

First, we assessed the protein levels of the NLRP3 inflammasome components to identify whether the NLRP3 inflammasome was altered in obese hearts. Mice were fed the ND or HFD for 24 weeks. NLRP3 protein level was intensely elevated in the myocardial tissue of obese mice (Figure 1A and 1B). Similarly, immunofluorescence staining confirmed the elevation of NLRP3 protein in obese hearts, specifically a significant increase of cytoplasmic NLRP3 (Figure 1C). Increased protein expression of other NLRP3 inflammasome components, like ASC and active caspase‐1 p20 was also detected in myocardial tissues of obese mice than ND mice (Figure 1A and 1B). Furthermore, to investigate the impact of chronic obesity on the NLRP3 inflammasome activation, we examined the protein levels of NLRP3 inflammasome in heart tissues of mice fed HFD for different durations. HFD mice exhibited a distinct obese phenotype. NLRP3 protein was elevated after 4 weeks of HFD feeding and then increased time‐dependently until 20 weeks but not after 24 weeks (Figure S1A through S1C). ASC protein levels did not change up to 16 weeks of HFD feeding but increased after 20 weeks of HFD feeding (Figure S1A and S1D). The protein level of active caspase‐1 p20 was mildly elevated after 16 weeks of HFD feeding, significantly elevated at 20 weeks, and did not decline at 24 weeks (Figure S1A and S1E). We further examined the expression level of proinflammatory factors IL‐1β and IL‐18 in obese hearts. IL‐1β and IL‐18 protein, detected by ELISA and immunoblotting, were significantly elevated in the myocardial tissue of HFD mice than in ND mice (Figure 1A through 1E). Notably, IL‐1β and IL‐18 protein expression was mildly elevated at around 16 weeks of HFD consumption, significantly elevated at 20 weeks, and maintained at high levels until 24 weeks (Figure S1A, S1F, and S1G). Immunohistochemical staining confirmed a clear increase in IL‐1β protein levels in the obese hearts (Figure 1F). Immunofluorescence results showed that macrophages in mouse myocardial tissue did not increase in response to chronic obesity stimuli (Figure S1H and S1I). These results indicated that prolonged obesity stimulation induced NLRP3 inflammasome activation in myocardial tissue.

To mimic obesity cardiomyopathy in vitro, NRVMs were incubated with BSA plus palmitic acid (PA) for 24 hours. Cytoplasmic ASC protein was significantly increased in BSA+PA‐cultured NRVMs (Figure S1J). Consistent with our findings in obese mice, NLRP3 inflammasome components, including NLRP3, ASC, and active caspase‐1 p20, were markedly increased in BSA+PA‐treated NRVMs compared with BSA‐treated NRVMs (Figure 1G and Figure S1K). NLRP3 protein in BSA‐treated NRVMs was mainly expressed in the nucleus with little expression in the cytoplasm, whereas NLRP3 protein was abundantly expressed in the cytoplasm of BSA+PA‐treated NRVMs (Figure 1H). Immunoblotting confirmed that IL‐1β and IL‐18 protein were elevated after BSA+PA treatment (Figure 1G and Figure S1K). Overall, the results suggested that NLRP3 inflammasome was activated in obesity cardiomyopathy.

### Genetic Disruption of NLRP3 Ameliorates Obesity‐Induced Cardiomyopathy

To identify the role of the NLRP3 inflammasome in obese hearts, NLRP3^‐/‐^ and wild‐type (WT) mice were fed HFD for 24 weeks to induce obesity cardiomyopathy. Deletion of NLRP3 protein in myocardial tissues from NLRP3^‐/‐^ mice was verified by immunoblotting (Figure S2A). In comparison with mice receiving ND feeding, HFD mice develop a distinct obesity phenotype, as evidenced by significant weight gain and elevated fasting blood glucose (FBG) (Figure S2B and S2C). However, there was no difference in serum triglycerides between HFD and ND mice (Figure S2D). Serum triglycerides and FBG levels did not differ among HFD mice with or without NLRP3 deletion (Figure S2C and S2D). Previous studies have disclosed that weight gain is accompanied by NLRP3 inflammasome activation.^3^ NLRP3 deletion had no effect on body weight in ND mice. After 16 weeks of HFD feeding, a mild reduction in body weight was observed in NLRP3^‐/‐^ mice compared with WT mice. NLRP3^‐/‐^ mice had a clearly lower body weights than WT mice after 20 and 24 weeks of HFD feeding (Figure S2B).

**Figure 2:**
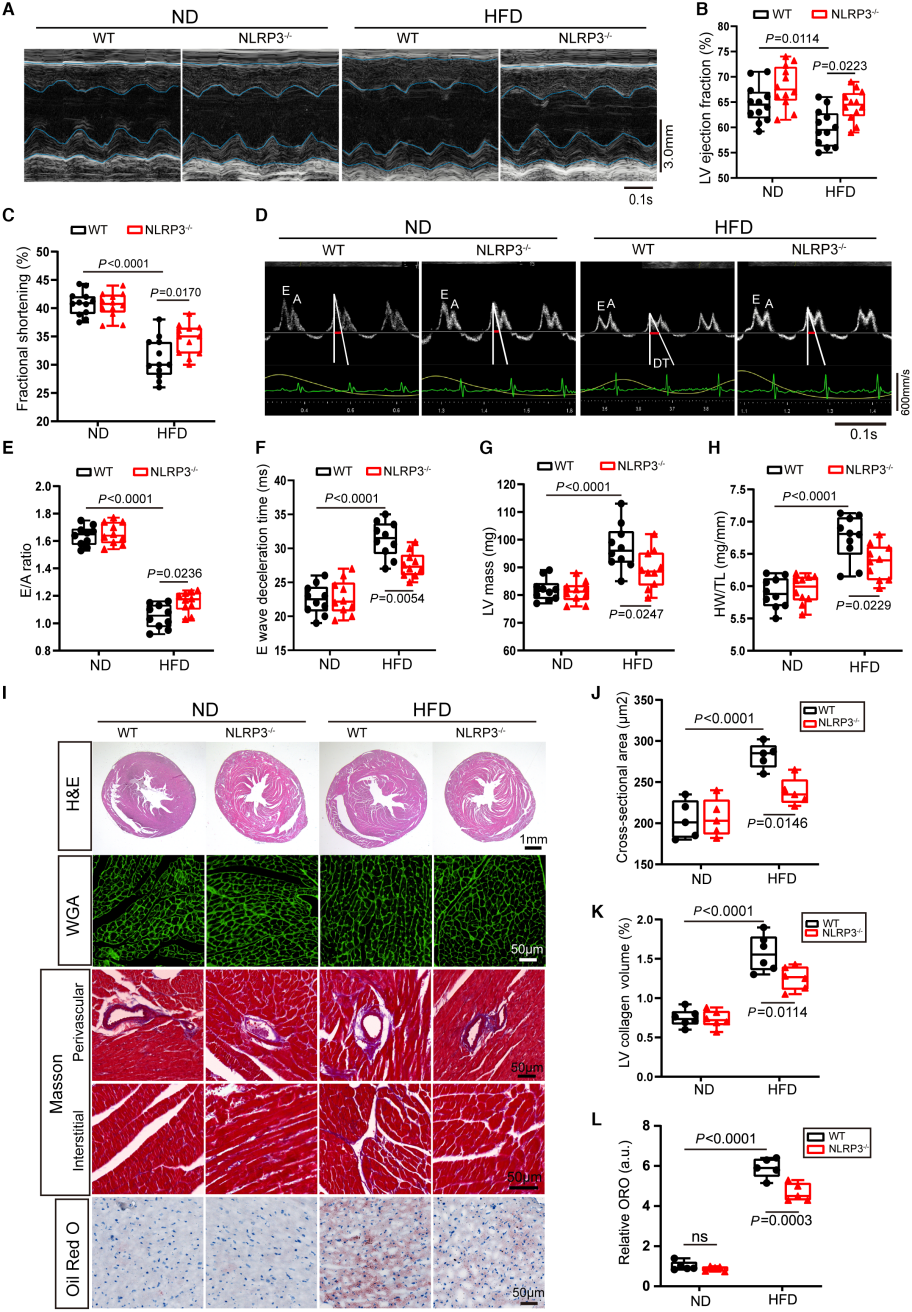
Genetic disruption of NLRP3 ameliorates obesity‐induced cardiomyopathy. **A**, Representative M‐mode echocardiographic images of left ventricle from WT or NLRP3^‐/‐^ mice subjected to ND or HFD feeding for 24 wks. **B**, **C**, LV ejection fraction and fractional shortening were measured by echocardiography (n=12 per group). **D**, **E**, Representative images of transmitral flow obtained by Doppler echocardiography and ratio between mitral E wave and A wave in WT or NLRP3^‐/‐^ mice subjected to ND or HFD feeding for 24 wks. E wave DT is indicated by red bars (n=10 per group). **F**, E wave DT, a marker of diastolic function, evaluated by transmitral flow Doppler echocardiography in indicated mice (n=10 per group). **G**, LV mass was measured by echocardiography in WT or NLRP3^‐/‐^ mice subjected to ND or HFD feeding for 24 wks (n=10 per group). **H**, Heart weight normalized by tibia length, a marker of cardiac hypertrophy. **I**, Representative images of cardiac H&E, WGA staining, Oil Red O staining and Masson trichrome staining in perivascular and interstitial area. **J**, Quantification of cardiomyocyte cross‐sectional area by WGA in indicated hearts (n=5 per group). **K**, Quantification of left ventricle collagen volume by Masson trichrome staining (n=6 per group). **L**, Quantification of myocardial lipid accumulation by Oil Red O staining (n=5 per group). DT indicates deceleration time; H&E, hematoxylin and eosin; HFD, high‐fat diet; LV, left ventricular; ND, normal diet; ns, not significant; NLRP3, nucleotide‐binding oligomerization domain‐like receptor 3; ORO, Oil Red O; WGA, wheat germ agglutinin; and WT, wild type.

Cardiac function was examined by echocardiography after 24 weeks of ND or HFD feeding. Obese mice developed left ventricular (LV) systolic dysfunction as evidenced by decreased LV fractional shortening and a mild decline in LV ejection fraction, which was partially ameliorated by NLRP3 deletion (Figure 2A through 2C). HFD induced severe cardiac diastolic dysfunction as evidenced by an extremely decreased early to late diastolic transmitral flow velocity (E/A ratio), significantly lower transmitral flow (E wave), and a longer deceleration time of E wave. NLRP3 deletion attenuated obesity‐induced cardiac diastolic dysfunction (Figure 2D through 2F, Figure S2E). These results suggested that genetic disruption of NLRP3 improved HFD‐induced cardiac systolic and diastolic function.

Furthermore, we assessed the effect of *NLRP3* deletion on cardiac remodeling in obese mice. Obese hearts developed myocardial hypertrophy as evidenced by increased heart size, heart mass, and the LV weight to tibia length ratio (HW/TL ratio), which was significantly mitigated by NLRP3 deletion (Figure 2G through 2I). Wheat germ agglutinin staining showed that NLRP3 deletion attenuated the increase in cardiomyocyte cross‐sectional area due to chronic obesity stimuli (Figure 2I and 2J). Likewise, NLRP3 deletion mitigated cardiac fibrosis, as assessed by reduced LV collagen volume in obese hearts (Figure 2I and 2K). Furthermore, real‐time PCR results suggested that NLRP3 deletion suppressed the expression of cardiac hypertrophy‐related genes (*ANP*, *BNP*, and *Myh7*) and cardiac fibrosis‐related genes (*Col1a1* and *CTGF*) in obese hearts (Figure S2F and S2G). Immunoblotting results further confirmed that NLRP3 deletion ameliorated cardiac remodeling, as evidenced by decreased protein levels of BNP, collagen I, and CTGF (Figure S2H and S2I). Cardiac lipotoxicity promotes progression of obesity‐induced heart failure.^17^ Oil red O staining results indicated that obese mice exhibited increased intramyocardial lipid contents, and NLRP3 deletion alleviated lipid accumulation (Figure 2I and 2L). Next, we examined the gene expression related to fatty acid metabolism by real‐time PCR. CD36 was considered a key regulatory protein for fatty acid uptake and transport in cardiomyocytes. The mRNA level of CD36 was significantly elevated in myocardial tissues of obese mice, and NLRP3 deletion did not affect its expression. The mRNA expression of Cpt1b in the obese hearts was unchanged, but the mRNA expression of other genes related to FAO (*Acadl* and *Acadvl*) was elevated. Intriguingly, NLRP3 deletion increased mRNA levels of genes involved in FAO (*Cpt1b*, *Acadl*, and *Acadvl*) in myocardial tissues of obese mice (Figure S2J). As expected, NLRP3 deletion reduced protein levels of ASC, active caspase‐1 p20, IL‐1β, and IL‐18 in the hearts of obese mice (Figure S2K). Furthermore, ELISA results also confirmed that NLRP3 deletion severely reduced protein level of proinflammatory cytokine IL‐1β and IL‐18 in obese hearts (Figure S2L and S2M). In summary, these results suggest that genetic disruption of NLRP3 improved HFD‐induced cardiac dysfunction, pathological cardiac remodeling, and lipid accumulation.

### NLRP3 Inflammasome Inhibitor MCC950 Ameliorates Obesity Cardiomyopathy

**Figure 3:**
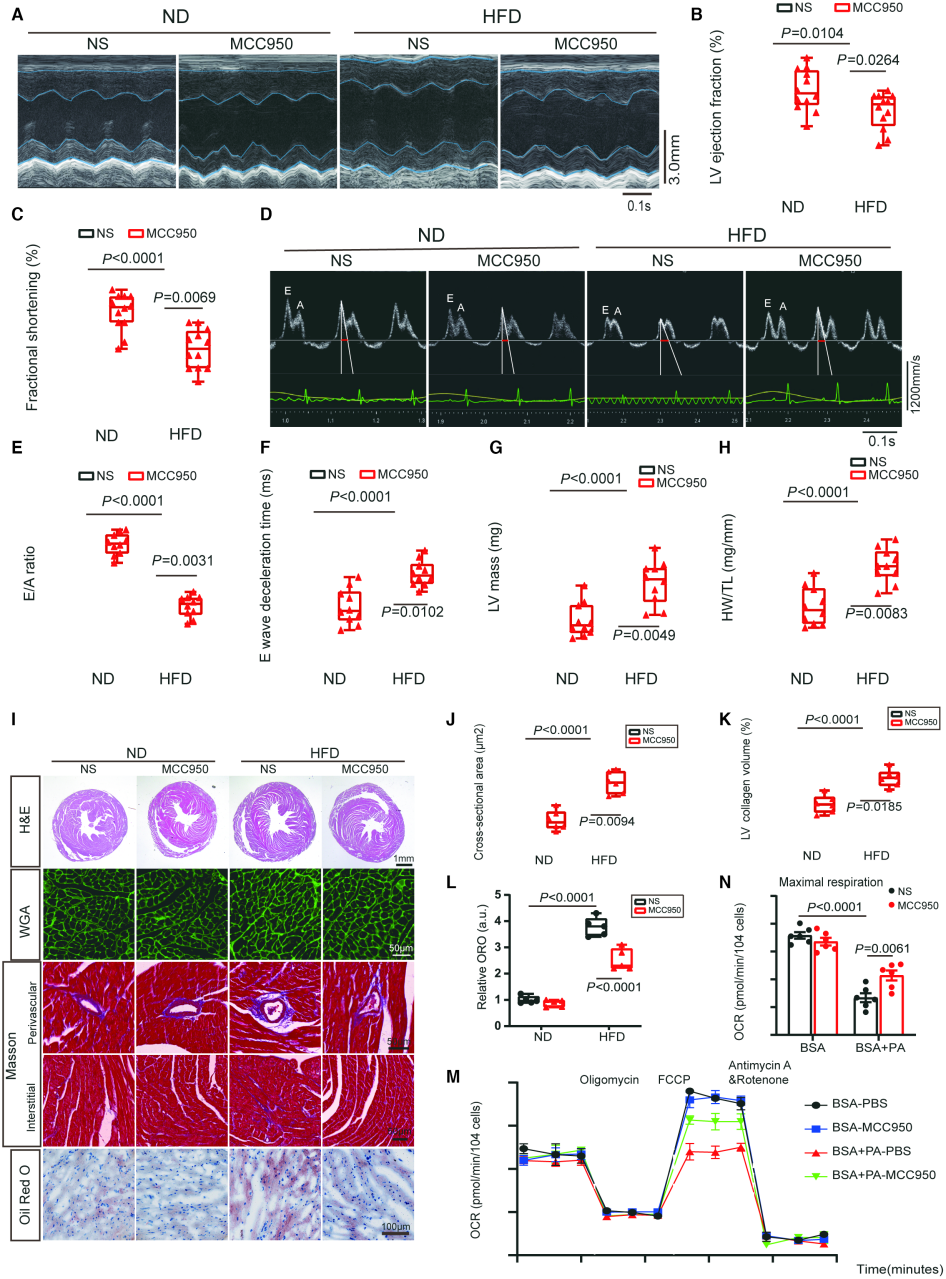
NLRP3 inflammasome inhibitor MCC950 ameliorates obesity cardiomyopathy. **A** through **L**, Wild‐type mice treated with NS or MCC950 for 12 wks were subjected to ND or HFD feeding for 24 wks. **A**, Representative M‐mode echocardiographic images of left ventricle from the indicated mice. **B**, **C**, LV ejection fraction and fractional shortening were measured by echocardiography (n=12 per group). **D**, **E**, Representative images of transmitral flow obtained by Doppler echocardiography and ratio between mitral E wave and A wave. E wave DT is indicated by red bars (n=10 per group). **F**, E wave DT, a marker of diastolic function, evaluated by transmitral flow Doppler echocardiography in indicated mice (n=10 per group). **G**, LV mass was measured by echocardiography in indicated mice subjected to NS or MCC950 for 12 wks (n=10 per group). **H**, Heart weight normalized by tibia length, a marker of cardiac hypertrophy (n=10 per group). **I**, Representative images of cardiac H&E, WGA staining, Oil Red O staining and Masson trichrome staining in perivascular and interstitial area. **J**, Quantification of cardiomyocyte cross‐sectional area by WGA in indicated hearts (n=6 per group). **K**, Quantification of LV collagen volume by Masson trichrome staining (n=6 per group). **L**, Quantification of myocardial lipid accumulation by Oil Red O staining (n=6 per group). **M**, **N**, Mitochondrial oxidative capacity was measured in real time after MCC950 treatment or BSA+PA incubation in NRVMs. Quantification of maximal respiration in (**L**) (n=6 independent experiments). DT indicates deceleration time; FCCP, Trifluoromethoxy carbonylcyanide phenylhydrazone; H&E, hematoxylin and eosin; HFD, high‐fat diet; BSA, Bovine Serum Albumin; HW/TL, heart weight/tibia length ratio; ND, normal diet; NLRP3, nucleotide‐binding oligomerization domain‐like receptor 3; NRVM, neonatal rat ventricular myocyte; NS, normal saline; OCR, oxygen consumption rate; ORO, Oil Red O; PA, palmitic acid; and WGA, wheat germ agglutinin.

NLRP3 inflammasome in obese hearts were unchanged after 12 weeks of HFD consumption but appeared elevated at approximately 16 weeks (Figure S1A). To further verify the role of NLRP3 inflammasome in obese cardiomyopathy, NLRP3 inflammasome inhibitor MCC950 was administered intraperitoneally to obese mice after 12 weeks of HFD consumption at a dose of 10 mg/kg and day for 12 weeks (Figure S3A). Although MCC950 did not affect serum triglycerides, it reduced body weight and FBG in obese mice after 24 weeks of HFD feeding (Figure S3B and S3D). MCC950 had minimal effect on left ventricle cardiac contraction in ND mice but improved systolic dysfunction in obese mice, as shown by higher LV ejection fraction and fractional shortening (Figure 3A through 3C). Similarly, MCC950 significantly improved HFD‐induced cardiac diastolic dysfunction, as evidenced by elevated E wave, higher E/A ratio, and a decline in E wave deceleration time (Figure 3D through 3F, Figure S3E).

We also evaluated the effect of MCC950 on cardiac remodeling in obese mice. MCC950 attenuated HFD‐induced myocardial hypertrophy, as showed by reductions in heart size, LV mass, and HW/TL (Figure 3G through 3I). Additionally, injection of MCC950 reduced the cross‐sectional area of cardiomyocytes in obese mice. Masson trichrome staining showed that MCC950 mitigated cardiac fibrosis in obese hearts, as assessed by reduced LV collagen volume (Figure 3I through 3K). Real‐time PCR results suggested that MCC950 suppressed the expression of genes involved in myocardial hypertrophy and cardiac fibrosis in the obese hearts (Figure S3F and S3G). Immunoblotting results further confirmed that MCC950 improved cardiac remodeling as evidenced by decreased expression of the hypertrophy marker protein BNP and the fibrosis‐associated proteins collagen I and CTGF (Figure S3H through S3K). As expected, MCC950 reduced protein expression of active caspase‐1 p20, IL‐1β, and IL‐18 in the obese hearts (Figure S3H, S3L and S3M). Although MCC950 was thought to specifically inhibit the assembly of NLRP3 inflammasomes, thereby hindering the production of IL‐1β and IL‐18. Interestingly, we observed that MCC950 reduced the protein level of NLRP3 and ASC in obese hearts (Figure S3H). We next evaluated the effects of MCC950 on cardiac lipotoxicity in obese mice. Oil red O staining indicated that MCC950 attenuated lipid accumulation in obese hearts (Figure 3I and 3L). MCC950 treatment alleviated lipid storage in BSA+PA incubated NRVMs, as evidenced by a decline in the number and volume of intracellular lipid droplets (Figure S3N). Although injection of MCC950 did not alter CD36 gene expression, it increased the expression of FAO‐related genes (*Cpt1b*, *Acadl*, and *Acadvl*) (Figure S3O). Moreover, MCC950 restored maximal oxygen consumption rate after BSA+PA treatment. MCC950 ameliorated palmitic acid‐induced mitochondrial dysfunction in NRVMs (Figure 3M and 3N). These results suggested that NLRP3 inflammasome inhibition with MCC950 mitigated obesity cardiomyopathy.

### MCC950 Inhibits NF‐κB Activation‐Mediated Priming of NLRP3 Inflammasome

**Figure 4:**
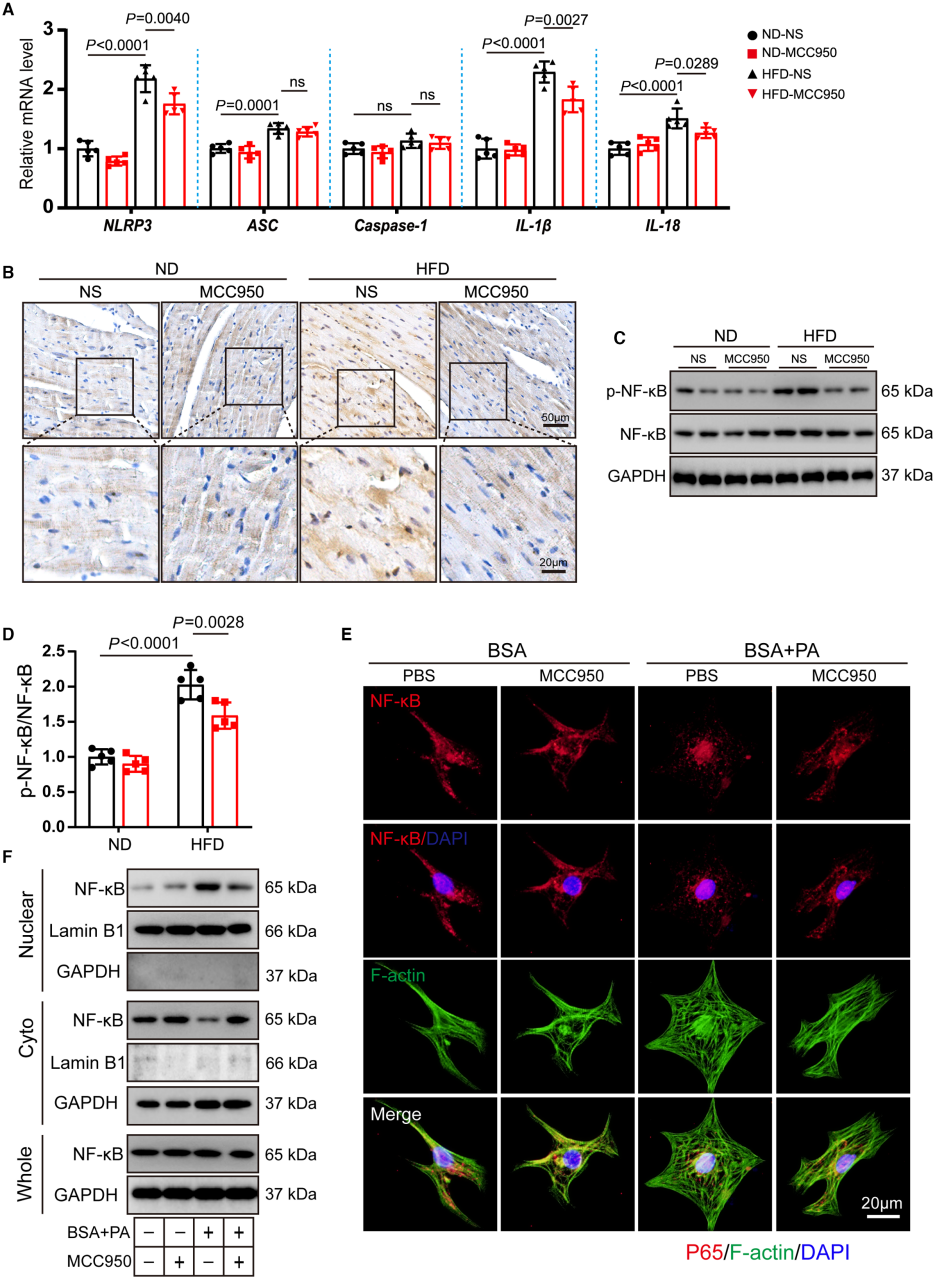
MCC950 inhibits NF‐κB activation‐mediated priming of NLRP3 inflammasome. **A** through **D**, Wild‐type mice treated with NS or MCC950 for 12 wks were subjected to ND or HFD feeding for 24 wks. **A**, qRT‐PCR detection of indicated genes related to NLRP3 inflammasome components and the proinflammatory cytokine IL‐1β and IL‐18 in hearts from indicated mice (n=5 per group). **B**, Immunohistochemical staining for NF‐κB in indicated murine heart. **C**, **D**, Representative images of immunoblot and quantitative analysis of NF‐κB in heart tissues from indicated mice. **E** through **F**, NRVMs treated with NS or MCC950 were incubated with BSA or BSA+PA medium for 24 h. **E**, Representative images of immunofluorescence staining of NF‐κB (red) and F‐Actin (green) in NRVMs. **F**, Immunoblotting analysis of NF‐κB protein in whole‐cell lysates and cytoplasmic and nuclear components in the indicated NRVMs. HFD indicates high‐fat diet; IL, interleukin; ND, normal diet; NF‐κB, nuclear factor kappa B; NLRP3, nucleotide‐binding oligomerization domain‐like receptor 3; NRVM, neonatal rat ventricular myocyte; ns, not significant; NS, normal saline; p‐NF‐κB, phosphorylated nuclear factor kappa B; PA, palmitic acid; and qRT‐PCR, quantitative real‐time polymerase chain reaction.

NF‐κB is a key regulator of priming signal of NLRP3 inflammasome activation, which induces the transcription of NLRP3 and IL‐1β^9^. Immunoblotting results showed that MCC950 reduced the protein level of NLRP3 and ASC in obese hearts (Figure S3H). To further investigate whether MCC950 attenuates the priming step of NLRP3 inflammasome, we examined the expression and activation of NF‐κB protein, as well as the transcription levels of genes related to NLRP3 inflammasome components. The mRNA levels of *NLRP3*, *IL‐1β*, and *IL18* were elevated in obese hearts, which was partially attenuated by MCC950. However, MCC950 failed to reduce obesity‐induced elevated *ASC* mRNA levels in obese heart. HFD and MCC950 had no effect on *caspase‐1* gene expression (Figure 4A). The active NF‐κB promotes the transcription of NLRP3 and IL‐1β in the nucleus. Immunohistochemistry staining confirmed that HFD induced NF‐κB nuclear localization, which was partially inhibited by MCC950 (Figure 4B). Immunoblotting also confirmed that HFD induced aberrant upregulation of the NF‐κB signaling pathway as evidenced by elevated protein levels of p‐NF‐κB, which was alleviated by MCC950 (Figure 4C and 4D). Furthermore, we explored the effect of MCC950 on the activation of the NF‐κB signaling pathway in vitro. BSA+PA treatment promoted cardiomyocyte hypertrophy as reflected by increased cardiomyocyte surface area, which was mitigated by MCC950 (Figure 4E and Figure S4A). Immunofluorescence results confirmed that MCC950 partially inhibited BSA+PA treatment‐induced elevation of NF‐κB protein in NRVMs (Figure 4E). In line with our findings in obese mice, MCC950 attenuated BSA+PA‐induced abnormal activation of NF‐κB signaling pathway in cardiomyocytes, as evidenced by decreased NF‐κB protein in the nucleus (Figure 4F). Real‐time PCR results suggested that MCC950 reduced the mRNA levels of NLRP3, ASC, IL‐1β, and IL‐18 in BSA+PA incubated NRVMs. However, MCC950 did not alter gene expression of caspase‐1 (Figure S4B). To investigate whether MCC950 directly affected NF‐κB activation in obese hearts, we examined the expression and localization of NF‐κB in myocardial tissues of NLRP3^‐/‐^ mice after 24 weeks of HFD. Immunoblotting results showed that p‐NF‐κB protein was significantly reduced in the heart of NLRP3^‐/‐^ obese mice than WT obese mice (Figure S4C and S4D). Taken together, either MCC950 or NLRP3 deletion prevented abnormal NF‐κB signaling pathway activation. We speculated that MCC950 failed to regulate NF‐κB activation directly, but it may ameliorate the aberrant activation of the NF‐κB signaling pathway by inhibiting production of proinflammatory cytokines IL‐1β and IL‐18.

### NLRP3 Deletion Abrogates TXNIP Overexpression‐Induced Exacerbation of Heart Failure in Obese Mice

**Figure 5:**
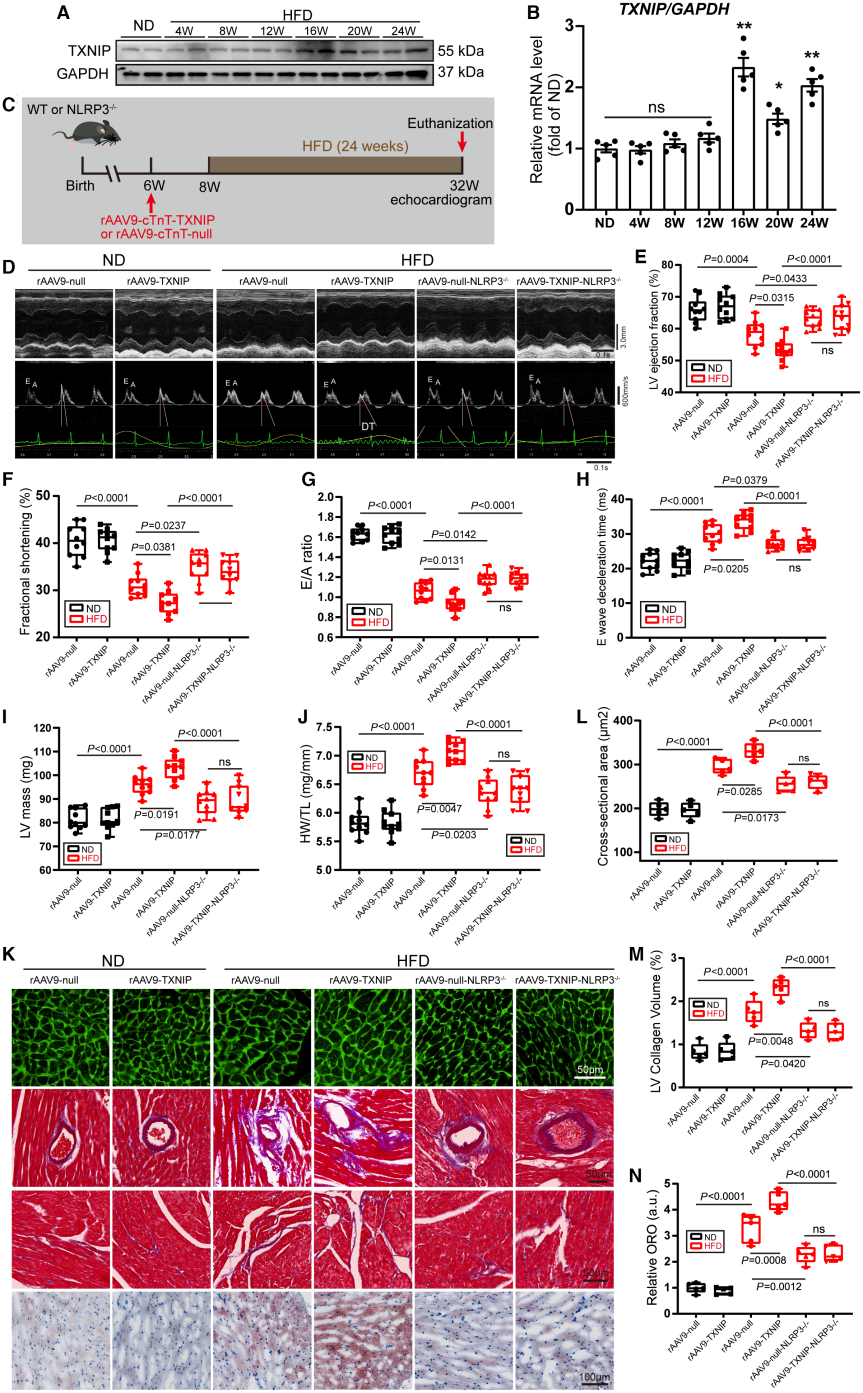
NLRP3 deletion abrogates TXNIP overexpression‐induced exacerbation of heart failure in obese hearts. **A**, Representative immunoblots of TXNIP in the whole‐cell lysate of the heart at different time points following HFD consumption in mice. **B**, qRT‐PCR detection of TXNIP in hearts of mice fed an ND or an HFD for different durations (n=5 per group). **C**, Protocol and schematics of establishment of TXNIP overexpressing mouse model in WT or NLRP3^‐/‐^ mice subjected to ND or HFD feeding for 24 wks. **D** through **N**, WT or NLRP3^‐/‐^ mice received rAAV9‐cTnT‐null or rAAV9‐cTnT‐TXNIP were subjected to ND or HFD feeding for 24 wks. **D**, Representative images of M‐model echocardiography (upper) and transmitral flow obtained by Doppler echocardiography (lower). E wave deceleration time is indicated by red bars. **E**, **F**, LV ejection fraction (**E**) and fractional shortening (**F**) were measured by echocardiography in indicated mice (n=10 per group). **G**, **H**, Histogram of ratio between mitral E wave and A wave and E wave deceleration time, evaluated by transmitral flow Doppler echocardiography in indicated mice (n=10 per group). **I**, **J**, Histogram of LV mass (**I**) and HW/TL (**J**) in indicated mice (n=10 per group). **K**, Representative images of cardiac WGA staining, Oil Red O staining and Masson trichrome staining in perivascular and interstitial area (n=10 per group). **L**, Quantification of cardiomyocyte cross‐sectional area by WGA (n=5 per group). **M**, Quantification of LV collagen volume in interstitial area by Masson trichrome staining (n=5 per group). **N**, Quantification of myocardial lipid accumulation by Oil Red O staining (n=5 per group). cTnT indicates cardiac troponin T; HFD, high‐fat diet; HW/TL, heart weight/tibia length ratio; LV, left ventricular; ND, normal diet; ns, not significant; NLRP3, nucleotide‐binding oligomerization domain‐like receptor 3; ORO, Oil Red O; qRT‐PCR, quantitative real‐time polymerase chain reaction; rAAV9, recombinant adeno‐associated virus serotype 9 encoding TXNIP; TXNIP, thioredoxin‐interacting protein; WGA, wheat germ agglutinin; and WT, wild type.

In response to ROS, TXNIP segregates from TRX and binds to NLRP3 thereby leading to the assembly of NLRP3 inflammasome, which is an important mode of NLRP3 inflammasome activation.^19^ Immunoblotting and real‐time PCR results showed that TXNIP mRNA and protein levels began to increase after 16 weeks of HFD feeding and remained elevated until 24 weeks (Figure 5A and 5B). To investigate whether TXNIP/NLRP3 inflammasome activation contributes to obesity‐induced cardiomyopathy, we specifically overexpressed TXNIP protein in cardiomyocytes by delivering recombinant adeno‐associated virus serotype 9 encoding TXNIP (rAAV9‐cTnT‐TXNIP) into 6‐week‐old WT or NLRP3^‐/‐^ mice, with rAAV9‐cTnT‐null as a control (Figure 5C). Two weeks later, the efficiency of TXNIP overexpression was verified by immunoblotting (Figure S5A). Obese mice had significant weight gain and higher FBG than ND mice. Notably, TXNIP overexpression had no effect on body weight but increased FBG levels in obese mice. However, weight loss and decreased FBG levels were observed in NLRP3^‐/‐^ obese mice with or without TXNIP overexpression (Figure S5B and S5C). Consistent with our previous findings, obese mice exhibited cardiac systolic and diastolic dysfunction, as evidenced by reduced LV ejection fraction and fractional shortening, prolonged E wave deceleration time, and decreased E wave and E/A ratio. Importantly, TXNIP overexpression aggravated HFD‐induced cardiac diastolic and systolic dysfunction, which was completely alleviated by NLRP3 deletion (Figure 5D through 5H, Figure S5D).

We also evaluated the effect of TXNIP overexpression on cardiac remodeling and lipid accumulation in obese mice. Obese mice also exhibited myocardial hypertrophy as evidenced by increased LV mass, HW/TL, and cardiomyocyte cross‐sectional area. Notably, TXNIP overexpression exacerbated obesity‐induced cardiac hypertrophy. NLRP3 deletion attenuated cardiac hypertrophy in obese mice with or without TXNIP overexpression (Figure 5I through 5L). Similarly, Masson trichrome staining indicated that NLRP3 deletion abrogated TXNIP overexpression‐induced worsening of cardiac fibrosis in obese hearts (Figure 5K and 5M). Consistently, immunoblotting further demonstrated that NLRP3 deletion attenuated the aggravation of cardiac hypertrophy and fibrosis caused by TXNIP overexpression (Figure S5E). Oil Red staining results indicated that TXNIP overexpression exacerbated lipid accumulation in obese hearts, which could be alleviated by NLRP3 deletion (Figure 5K and 5N). These results indicated that NLRP3 deletion abrogated deterioration of cardiac remodeling and lipid accumulation due to TXNIP expression in obese hearts.

Next, we explored the mechanism of TXNIP/NLRP3 inflammasome activation in obese hearts. Immunofluorescence staining indicated elevated protein levels of NLRP3 and TXNIP in obese hearts. HFD increased colocalization of cytosolic NLRP3 and TXNIP in myocardial tissue. TXNIP overexpression further increased colocalization of NLRP3 and TXNIP in obese hearts (Figure S5F). To explore whether TXNIP/NLRP3 inflammasome activation contributed to production of mature IL‐1β and IL‐18, protein levels of IL‐1β and IL‐18 were detected by ELISA. Although TXNIP overexpression did not alter IL‐1β and IL‐18 levels in ND mice, it significantly increased the protein expression of IL‐1β and IL‐18 in obese hearts (Figure S5G and S5H). These results indicated that TXNIP overexpression aggravated IL‐1β and IL‐18 production due to NLRP3 inflammasome activation. An endogenous coimmunoprecipitation assay using TXNIP antibody showed that HFD enhanced the interaction of TXNIP and NLRP3 to form NLRP3 inflammasome. Importantly, we found that the TXNIP‐NLRLP3 complex was markedly enriched in the hearts of obese mice with TXNIP overexpression compared with rAAV9‐null obese mice (Figure S5I). Immunoblotting results showed that TXNIP overexpression exacerbated HFD‐induced IL‐1β and IL‐18 production in obese hearts. NLRP3 deletion significantly reduced production of mature IL‐1β and IL‐18 in obese mice with or without TXNIP overexpression (Figure S5J). Collectively, NLRP3 deletion abrogated TXNIP overexpression‐induced exacerbation of heart failure in obese hearts.

### Inhibition of Mitochondrial ROS Ameliorates TXNIP/NLRP3 Inflammasome Activation and Improved Lipid Metabolism

**Figure 6:**
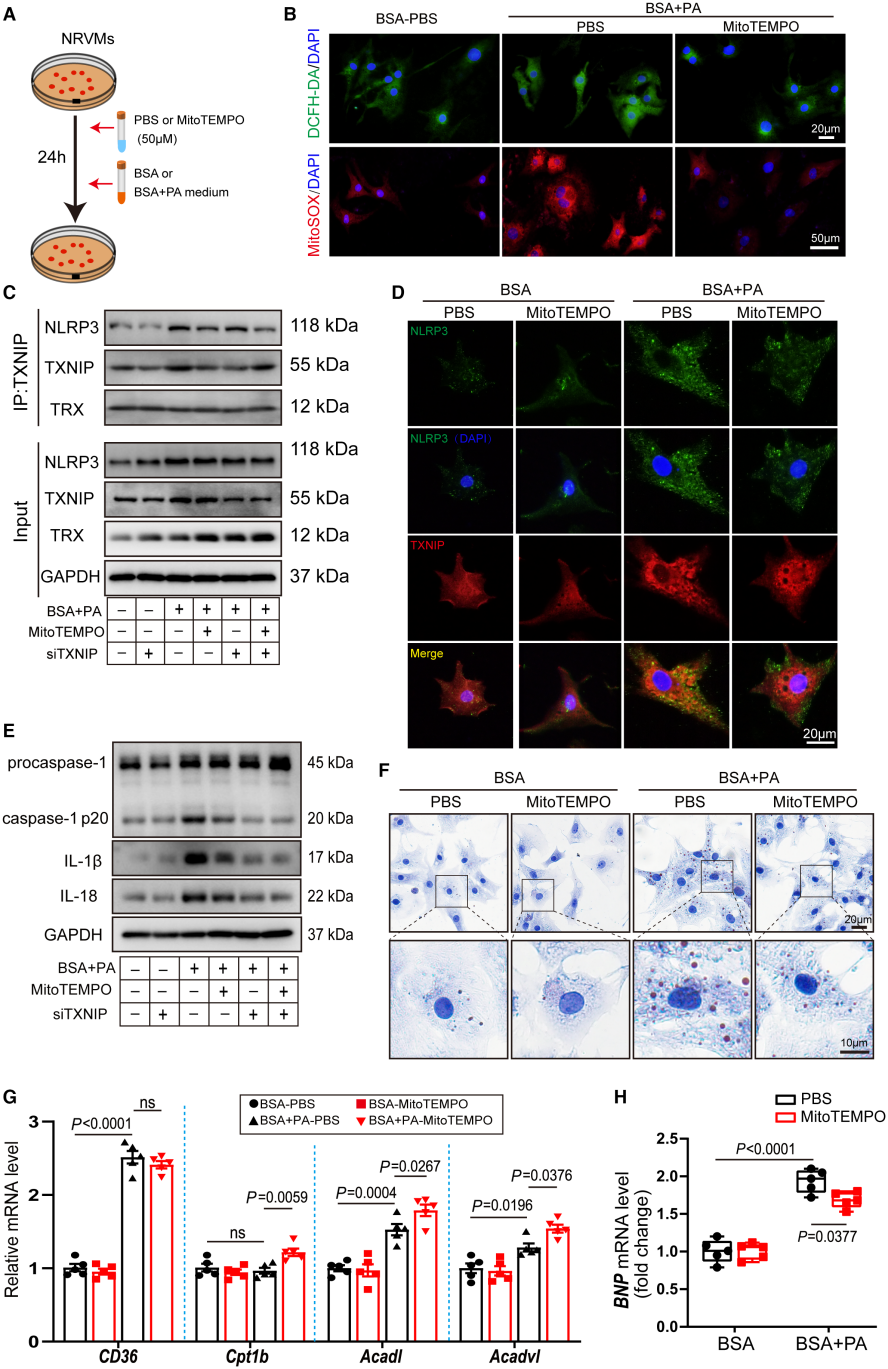
Inhibition of mitochondrial ROS ameliorates BSA+PA‐induced activation of TXNIP/NLRP3 inflammasome and impaired lipid metabolism. **A** through **H**, NRVMs treated with PBS or MitoTEMPO were incubated with BSA or BSA+PA medium for 24 h. **A**, Schematic diagram showing the procedure of NRVMs. **B**, Intracellular total ROS using DCFH‐DA staining (upper) and mitochondrial ROS using MitoSOX staining (lower) were determined in NRVMs. **C**, Cell lysates of NRVMs were immunoprecipitated with TXNIP antibody, and immunoblot assays were performed using NLRP3, TXNIP, and TRX antibodies. NRVMs were treated with siTXNIP to knockdown of TXNIP. **D**, Triple immunofluorescence staining for TXNIP (red), NLRP3 (green), and nuclei (DAPI, blue) was performed in indicated NRVMs. **E**, Representative immunoblots of ASC, procapase‐1, cleaved caspase‐1 p20, IL‐1β, and IL‐18 protein in NRVMs from indicated groups. **F**, Oil Red O staining of NRVMs from indicated groups. Red indicates lipid droplets, blue indicates nuclei. **G**, qRT‐PCR detection of indicated genes related to fatty acid transport genes *Cd36* and fatty acid oxidation genes *Cpt1b*, *Acadl*, and *Acadvl* in hearts from indicated mice (n=6 independent experiments). **H**, Expression level of hypertrophic marker gene BNP was determined by RT‐PCR and normalized to that of GAPDH (n=6 independent experiments). ASC indicates apoptosis‐associated speck like protein; BNP, B‐type natriuretic peptide; BSA, Bovine Serum Albumin; IL, interleukin; IP:TXNIP, immunoprecipitation:thioredoxin‐interacting protein; ns, not significant; NLRP3, nucleotide‐binding oligomerization domain‐like receptor 3; NRVM, neonatal rat ventricular myocyte; PA, palmitic acid; qRT‐PCR, quantitative real‐time polymerase chain reaction; and ROS, reactive oxygen species.

Previous studies have confirmed that ROS played an important role in TXNIP/NLRP3 inflammasome activation.^19^ Mitochondria is the primary site of ROS production in cardiomyocytes. To investigate the role of mitochondrial ROS in obesity cardiomyopathy, NRVMs were treated with 50 μmol MitoTEMPO for 24 hours. Meanwhile, BSA+PA treatment was used to mimic obesity cardiomyopathy in vitro (Figure 6A and Figure S6A). BSA+PA stimulation induced increased cytosolic ROS production detected by DCFH‐DA staining. MitoTEMPO markedly reduced mitochondrial ROS production, as accessed by MitoSOX Red probes, thereby attenuating cytoplasmic oxidative stress levels (Figure 6B, Figure S6B and S6C). To clarify the role of mitochondrial ROS in TXNIP/NLRP3 inflammasome activation in vitro, we treated NRVMs with siTXNIP or siCTR for 48 hours, followed by incubation with BSA+PA or BSA medium for 24 hours (Figure S6A). Immunoblotting and real‐time PCR results confirmed that TXNIP was successfully knocked down by siTXNIP in NRVMs (Figure S6D and S6E). BSA+PA treatment resulted in a noticeable decrease in cell viability. TXNIP knockdown exerted no effects on NRVMs viability in either BSA or BSA+PA medium (Figure S6F). Immunoblotting confirmed that BSA+PA treatment increased protein expression levels of NLRP3, TXNIP, and TRX in cardiomyocytes. TXNIP knockdown did not affect the protein level of TRX in BSA or BSA+PA medium. Importantly, endogenous coimmunoprecipitation assays demonstrated that MitoTEMPO decreased interaction of NLRP3 and TXNIP in NRVMs with or without TXNIP knockdown (Figure 6C). Immunofluorescence results showed a slight decrease in NLRP3 protein in the nucleus and a significant increase of the cytosolic NLRP3 upon BSA+PA stimulation. TXNIP protein was markedly increased in the BSA+PA‐treated NRVMs than in BSA‐treated NRVMs. MitoTEMPO significantly reduced the colocalization of NLRP3 and TXNIP upon BSA+PA stimulation (Figure 6D). We next examined the protein expression levels of NLRP3 inflammasome components and proinflammatory cytokines. The NLRP3 inflammasome was aberrantly activated upon BSA+PA stimulation, as evidenced by a significant elevation of active caspase‐1 p20, IL‐1β, and IL‐18, which was mitigated by MitoTEMPO. TXNIP knockdown production further inhibited NLRP3 inflammasome activation and IL‐1β and IL‐18 production (Figure 6E).

We also explored the role of MitoTEMPO or TXNIP knockdown on lipid metabolism in vitro. MitoTEMPO or siTXNIP reduced lipid accumulation in BSA+PA incubated NRVMs, as evidenced by a reduction in the size and number of lipid droplets inside cells (Figure 6F, Figure S6G). *CD36* mRNA level was significantly elevated in BSA+PA‐treated NRVMs, and MitoTEMPO or TXNIP knockdown did not affect its expression. Intriguingly, the expression of genes involved in FAO (*Acadl* and *Acadvl*) was mildly upregulated in BSA+PA‐treated NRVMs. *Cpt1b* mRNA level did not change upon BSA+PA stimulation. MitoTEMPO or TXNIP knockdown further enhanced the expression of FAO‐related genes (Figure 6G, Figure S6H). Additionally, real‐time PCR results suggested that MitoTEMPO or siTXNIP reduced BSA+PA‐induced heart failure in vitro, as evidence by a decline in *BNP* mRNA levels (Figure 6H, Figure S6I). Together, these results suggest that mitochondrial ROS inhibition by MitoTEMPO ameliorated BSA+PA‐induced activation of TXNIP/NLRP3 inflammasome and impaired lipid metabolism.

### MitoTEMPO Alleviates NLRP3 Inflammasome Activation and Rescues Obesity‐Induced Cardiomyopathy

**Figure 7:**
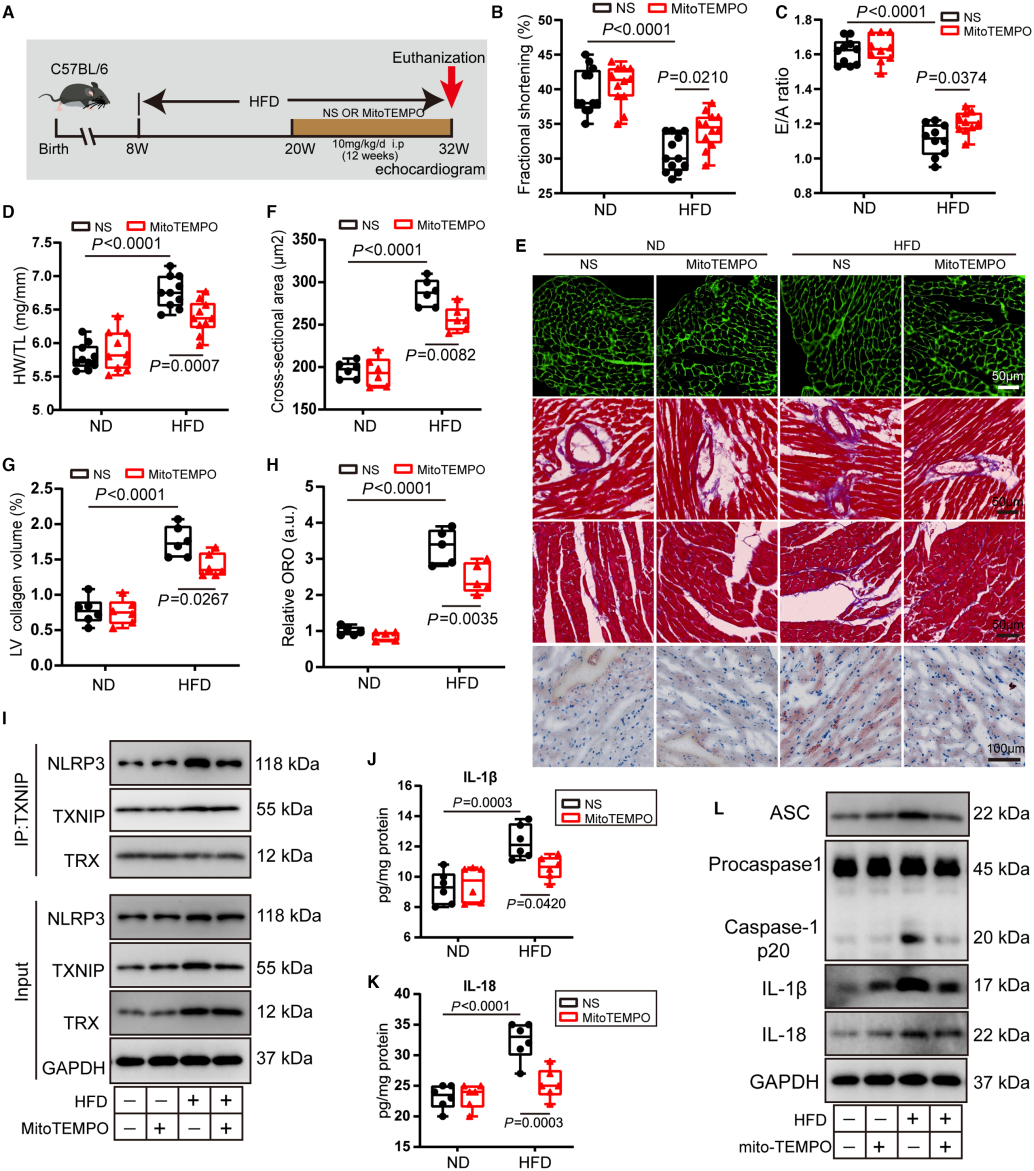
MitoTEMPO treatment alleviates NLRP3 inflammasome activation and rescues obesity‐induced cardiomyopathy. **A** through **L**, WT mice received NS or MitoTEMPO were subjected to ND or HFD feeding for 24 wks. **A**, Experimental schematic diagram showing the process of HFD‐induced obesity cardiomyopathy. After 12 wks of HFD feeding, MitoTEMPO was injected into obese mice at a dose of 10 mg/kg per day for 12 wks. **B**, **C**, LV fractional shortening (**B**) and ratio between mitral E wave and A wave (**C**) were measured by echocardiography in indicated mice (n=12 per group). **D**, Histogram of HW/TL in indicated mice. HW indicated wet weight (n=12 per group). **E**, Representative images of cardiac WGA staining, Oil Red O staining, and Masson trichrome staining in perivascular and interstitial area in indicated heart. **F**, Quantification of cardiomyocyte cross‐sectional area by WGA (n=6 per group). **G**, Quantification of LV collagen volume in interstitial area by Masson trichrome staining (n=6 per group). **H**, Quantification of myocardial lipid accumulation by Oil Red O staining (n=6 per group). **I**, Tissue lysate in indicated heart were immunoprecipitated with TXNIP antibody, and immunoblot assays were performed using NLRP3, TXNIP, and thioredoxin antibodies. **J**, **K**, Protein of IL‐1β (**J**) and IL‐18 (**K**) in the heart of WT mice fed an HFD or ND for 24 wks with or without MitoTEMPO treatment. IL‐1β and IL‐18 protein were detected by ELISA. Values were normalized to total protein level (n=6 per group). **L**, Representative immunoblots of ASC, procapase‐1, cleaved caspase‐1 p20, IL‐1β, and IL‐18 in the indicated heart. ASC indicates apoptosis‐associated speck like protein; HFD, high‐fat diet; HW/TL, heart weight/tibia length ratio; IL, interleukin; IP:TXNIP, immunoprecipitation:thioredoxin‐interacting protein; LV, left ventricular; ND, normal diet; ns, not significant; NLRP3, nucleotide‐binding oligomerization domain‐like receptor 3; NRVM, neonatal rat ventricular myocyte; ORO, Oil Red O; NS, normal saline; WGA, wheat germ agglutinin; and WT, wild type.

Our findings showed that NLRP3 inflammasome was activated after 16 weeks of HFD feeding and maintained until 24 weeks (Figure S1A). To investigate the role of MitoTEMPO in obesity cardiomyopathy in vivo, MitoTEMPO was injected into obese mice at a dose of 10 mg/kg per day for 12 weeks before aberrant NLRP3 inflammasome activation (Figure 7A). MitoTEMPO attenuated myocardial oxidative stress levels and reduced body weight in obese mice (Figure S7A and S7B). MitoTEMPO ameliorated systolic dysfunction in obese mice, as evidenced by elevated LV ejection fraction and fractional shortening (Figure 7B, Figure S7C and S7D). Similarly, MitoTEMPO improved HFD‐induced cardiac diastolic dysfunction, as evidenced by elevated E wave, higher E/A ratio, and a decline in E wave deceleration time (Figure 7C, Figure S7E and S7F).

We also evaluated the role of MitoTEMPO on cardiac remodeling in obese mice. MitoTEMPO attenuated obesity‐induced myocardial hypertrophy, as evidenced by reductions in LV mass and HW/WL ratio (Figure 7D, Figure S7G). Moreover, wheat germ agglutinin staining images indicated that MitoTEMPO reduced cardiomyocyte cross‐sectional area in obese mice (Figure 7E and 7F). MitoTEMPO also mitigated cardiac fibrosis in obese hearts, as assessed by reduced LV collagen volume (Figure 7E and 7G). Immunoblotting results further confirmed that MitoTEMPO attenuated myocardial hypertrophy and fibrosis as evidenced by decreased BNP and collagen I protein expression (Figure S7H through S7J). Oil Red O staining indicated that MitoTEMPO attenuated lipid accumulation in obese heart (Figure 7E and 7H). Although MitoTEMPO had no effect on *CD36* mRNA levels, it enhanced the expression of FAO‐related genes (*Cpt1b*, *Acadl*, and *Acadvl*) in obese hearts (Figure S7K). We hypothesized that enhanced FAO could alleviate obesity‐induced lipid accumulation. Importantly, endogenous coimmunoprecipitation assays demonstrated that MitoTEMPO did not affect the combination of TXNIP and TRX, but it markedly decreased interaction of NLRP3 and TXNIP in obese hearts (Figure 7I). Injection of MitoTEMPO reduced protein levels of IL‐1β and IL‐18 in myocardial tissue of obese mice (Figure 7J and 7K). Additionally, MitoTEMPO reduced protein levels of ASC, active caspase‐1 p20, IL‐1β, and IL‐18 in the hearts of obese mice (Figure 7L). In conclusion, these results indicate that MitoTEMPO alleviates obesity‐induced cardiac dysfunction and cardiac remodeling by inhibiting NLRP3 inflammasome activation.

## DISCUSSION

In the current study, we found that NLRP3 inflammasome activity is enhanced in cardiomyocytes of obese mice and PA‐incubated NRVMs. Aberrant NLRP3 inflammasome activation is an important pathogenetic mechanism in obesity cardiomyopathy. Inhibition of NLRP3 inflammasome by NLRP3 deletion or MCC950 mitigates obesity‐induced cardiac dysfunction, cardiac remodeling, and excessive lipid accumulation. Mechanistically, long‐term HFD consumption activates NF‐kB signaling pathway and increases mitochondrial ROS and TXNIP protein expression, resulting in enhanced NLRP3 inflammasome activity and massive IL‐1β and IL‐18 production. Our findings shed light on the pathogenesis of obesity cardiomyopathy and suggest that interventions that inhibit NLRP3 inflammasome activity are effective strategies for obesity cardiomyopathy.

Chronic obesity is strongly associated with heart failure, manifested by LV hypertrophy, cardiac fibrosis, and diastolic dysfunction, which eventually evolves into overt heart failure.^18^ Our study confirmed that NLRP3 inflammasome activation substantially contributed to obesity‐induced heart failure. NLRP3 deletion and MCC950 injection were used to inhibit myocardial NLRP3 inflammasome in obese mice. Impeding NLRP3 inflammasome significantly improved obesity‐induced cardiac remodeling and cardiac systolic and diastolic dysfunction. Of note, the improvement in cardiac diastolic function in this study was characterized specifically by an increase in the E/A ratio and E wave and a decline in the E wave deceleration time. NLRP3 inflammasome is abundantly expressed in macrophages.^9^ Macrophage NLRP3 inflammasome activation induces chronic inflammation in myocardial tissue, leading to mitochondrial damage and dysfunction in cardiomyocytes and exacerbating cardiac dysfunction.^12^, ^20^, ^21^ Furthermore, Pavillard and colleagues showed that impaired autophagy and oxidative stress induced the monocyte NLRP3 inflammasome activation after 16 weeks of HFD feeding, which promoted cardiac fibrosis and LV myocardial hypertrophy.^22^ In the present study, we found that CD68‐positive macrophages were not increased in obese hearts, and in vivo and in vitro experiments demonstrated that cardiomyocyte NLRP3 inflammasome was significantly increased in response to high‐fat stimulation. NLRP3 inflammasomes activation in cardiac nonimmune cells during the pathogenesis of heart failure has received increasing attention.^23^, ^24^ Previous studies found that cardiomyocyte mitochondrial DNA and mitochondrial hyperacetylation promoted NLRP3 inflammasome activation in the failing heart.^25^, ^26^ Moreover, cardiac fibroblasts NLRP3 inflammasomes activation exacerbated myocardial ischemia–reperfusion injury.^27^ In this study, we demonstrated that chronic HFD feeding induced NLRP3 inflammasomes activation in cardiomyocytes of obese heart. Furthermore, we found that the protein expression levels of NLRP3 inflammasome components such as NLRP3, ASC, and active caspase‐1 p20, as well as proinflammatory cytokines IL‐1β and IL‐18, were significantly elevated in BSA+PA‐treated NRVMs. Our results suggest that mitochondrial ROS‐mediated TXNIP/NLRP3 inflammasome activation in cardiomyocytes plays a critical role in the pathogenesis of obesity cardiomyopathy.

A previous study induced obesity cardiomyopathy in mice by sustained HFD feeding for 52 weeks. This study illustrated that NLRP3 deficiency ameliorated obesity‐induced cardiac diastolic dysfunction but had no effect on LV hypertrophy and myocardial fibrosis.^28^ Conversely, our findings suggest that aberrant NLRP3 inflammasome activation in cardiomyocytes is a major cause of HFD‐induced cardiac remodeling. Impeding NLRP3 inflammasome reduced cardiac lipid accumulation and attenuated cardiac hypertrophy and myocardial fibrosis. Notably, our study induced obesity cardiomyopathy in mice by HFD feeding for 24 weeks. We speculated that NLRP3 inflammasome inhibition ameliorates obesity‐induced cardiac remodeling; however, cardiac aging and sustained HFD for 52 weeks partially reduced its cardioprotective effects. In a recent study, Raggi and colleagues indicated that 16 weeks of HFD feeding resulted in elevated mRNA levels of NLRP3 and ASC in myocardial tissue. However, the protein levels of the proinflammatory factors IL‐1β and IL‐18 were unchanged in obese heart.^29^ Interestingly, our findings confirmed that IL‐1β and IL‐18 protein expression was mildly elevated at around 16 weeks of HFD consumption, significantly elevated at 20 weeks and maintained at high levels until 24 weeks. Although NLRP3 protein was increased as early as after 4 weeks of HFD feeding, protein expression of active caspase‐1 p20 was mildly elevated in obese mice only after 16 weeks of HFD feeding. In conclusion, the NLRP3 inflammasome activity as well as protein levels of IL‐1β and IL‐18 were not increased in obese heart during the early phase of the HFD feeding (12 weeks).

MCC950 is a specific inhibitor that selectively blocks the assembly of NLRP3 inflammasome but has no effect on the priming step in NLRP3 inflammasome activation.^30^, ^31^ Previous studies have demonstrated that MCC950 reduces NLRP3 inflammasome assembly by inhibiting NLRP3 protein activation and ASC oligomerization.^32^, ^33^ Of note, our findings indicated that MCC950 attenuated the priming step of NLRP3 inflammasome in the obese hearts by suppressing the NF‐κB signaling pathway activation. IL‐1β triggers nuclear factor‐κB activation by activating the IL‐1 receptor (IL‐1R) on the cell membrane.^9^, ^34^ In response to cellular injury and stress, mitochondrial DNA released from damaged mitochondria activates cytosolic TLR9, leading to activation of the NF‐κB signaling pathway.^35^ Moreover, we found that NLRP3 deletion also suppressed the NF‐κB signaling pathway in obese hearts. We speculated that MCC950 might also inhibit the activation of the NF‐κB signaling pathway by reducing activation of IL‐1R, or decreasing cytosolic mitochondrial DNA released from damaged mitochondria. Thus, MCC950 inhibits the priming step in NLRP3 inflammasome by regulating the activation of the NF‐κB signaling pathway.

TXNIP negatively regulates TRX activity and inhibits its antioxidant capacity. Moreover, TXNIP regulates serum glucose levels by inducing pancreatic β‐cell apoptosis and decreasing insulin production.^36^ A previous study indicated that TXNIP protein exacerbated cardiomyocyte apoptosis and cardiac dysfunction during myocardial ischemia–reperfusion injury by regulating the formation and clearance of autophagosomes.^37^ TXNIP inhibitor prevented cardiac hypertrophy by attenuating oxidative stress.^38^ However, another study has shown that TXNIP knockout exacerbates LV adverse myocardial remodeling after 8 weeks of pressure overload.^39^ It remains unclear whether TXNIP protein exacerbates myocardial hypertrophy and cardiac dysfunction. Recently, TXNIP knockdown ameliorated obesity cardiomyopathy by modulating mitochondrial dynamics and improving FAO.^40^ In the present study, we found elevated TXNIP protein expression in obese hearts. Our experimental results showed that chronic HFD feeding enhanced the interaction of TXNIP and NLRP3 to form NLRP3 inflammasome. TXNIP overexpression further enhanced TXNIP/NLRP3 inflammasome activation in the obese heart, leading to massive production of proinflammatory factors IL‐1β and IL‐18. Cardiomyocyte TXNIP overexpression aggravated obesity‐induced cardiac dysfunction, lipid accumulation, and cardiac remodeling. In addition, TXNIP knockdown significantly attenuated NLRP3 inflammasome activity and cardiac lipotoxicity in palmitic acid stimulated‐NRVMs.

Emerging evidence supports mitophagy protects the heart against HFD‐induced cardiomyopathy.^4^, ^17^ Impaired mitochondrial autophagy leads to reduced mitochondrial FAO. Obesity induces increased transcription of FAO‐related genes as a compensatory mechanism for insulin resistance. A recent study indicated that MCC950 restored fatty acid uptake and use by increasing the protein expression of CD36 and Cpt1b.^41^ We discovered that inhibition of NLRP3 inflammasome enhanced transcription of FAO‐related genes, thereby attenuating lipid accumulation in obese heart. However, NLRP3 inflammasome does not alter CD36 gene expression in obese heart. Our findings suggest that impeding NLRP3 inflammasome activity does not affect fatty acid uptake or transport but ameliorated mitochondrial dysfunction, leading to enhanced FAO.

### Limitations

Due to lack of human LV myocardial tissue, NLRP3 inflammasome components, TXNIP protein, and p‐NF‐κB were not detected in the hearts of patients with obesity. It is unclear whether NF‐κB signaling pathway activation contributes to the transcription of ASC in obese mice. If not, what are the reasons for the elevated mRNA and protein levels of ASC in obese hearts? Besides mitochondrial ROS‐mediated activation of TXNIP/NLRP3 inflammasome, we did not further explore other pathways leading to the activation of NLRP3 inflammasome. The role of NLRP3 inflammasome on lipid metabolism in the obese heart needs to be further investigated. Additionally, a cardiac‐specific NLRP3‐deletion mouse model needs to be constructed to investigate the role of cardiomyocyte NLRP3 inflammasome in obesity cardiomyopathy in vivo. Clinical trials have showed that MCC950 elicits elevations in serum liver enzymes levels.^30^ Liver function and serum liver enzymes should be examined after MCC950 treatment to evaluate the toxicity of MCC950.

A previous study showed that NLRP3 deletion did not affect blood pressure in mice with pressure load‐induced heart failure.^15^ The NLRP3 inhibitor MCC950 has also been shown to effectively lower blood pressure and attenuate renal inflammation, fibrosis and dysfunction in hypertensive mice.^42^ Furthermore, inhibition of NLRP3 inflammasome activation improves renal function in mice with acute kidney injury by upregulating mitochondrial autophagy.^43^ In this study, we did not measure blood pressure and renal function in mice. We were unable to assess the effects of NLRP3 inflammasome activation on hypertension and renal impairment in obese mice. Thus, blood pressure/renal function improvement may have been a potential confounding variable in this study.

## CONCLUSIONS

The current study confirmed that mitochondrial ROS‐mediated TXNIP/NLRP3 inflammasome activation in cardiomyocytes plays a critical role in the pathogenesis of obesity cardiomyopathy. Impeding NLRP3 inflammasome may be a potent therapeutic strategy for obesity‐associated cardiomyopathy.

## Sources of Funding

This work was supported by grants from the National Key R&D Program of China (2018YFC1311300); the Regional Innovation and Development Joint Fund of National Natural Science Foundation of China (No. U22A20269); the Key Project of the National Natural Science Foundation of China (No. 81530012); the National Natural Science Foundation of China (Nos. 81860080, 82170245, 82370284. and 81700254); and Fundamental Research Funds for the Central Universities (2042018kf1032).

## Disclosures

None.

## Supporting information

Data S1
Tables S1–S3
Figures S1–S7
References 44–49

## References

[1] Blüher M . Obesity: global epidemiology and pathogenesis. Nat Rev Endocrinol. 2019;15:288–298. doi: 10.1038/s41574-019-0176-8 30814686

[2] Chooi YC , Ding C , Magkos F . The epidemiology of obesity. Metabolism. 2019;92:6–10. doi: 10.1016/j.metabol.2018.09.005 30253139

[3] Ren J , Wu NN , Wang S , Sowers JR , Zhang Y . Obesity cardiomyopathy: evidence, mechanisms, and therapeutic implications. Physiol Rev. 2021;101:1745–1807. doi: 10.1152/physrev.00030.2020 33949876 PMC8422427

[4] Tong M , Saito T , Zhai P , Oka S‐I , Mizushima W , Nakamura M , Ikeda S , Shirakabe A , Sadoshima J . Alternative mitophagy protects the heart against obesity‐associated cardiomyopathy. Circ Res. 2021;129:1105–1121. doi: 10.1161/CIRCRESAHA.121.319377 34724805

[5] Mitter SS , Shah SJ , Thomas JD . A test in context: E/a and E/e′ to assess diastolic dysfunction and LV filling pressure. J Am Coll Cardiol. 2017;69:1451–1464. doi: 10.1016/j.jacc.2016.12.037 28302294

[6] Kishi S , Armstrong AC , Gidding SS , Colangelo LA , Venkatesh BA , Jacobs DR , Carr JJ , Terry JG , Liu K , Goff DC , et al. Association of obesity in early adulthood and middle age with incipient left ventricular dysfunction and structural remodeling: the CARDIA study (Coronary Artery Risk Development in Young Adults). JACC Heart Fail. 2014;2:500–508. doi: 10.1016/j.jchf.2014.03.001 25194290 PMC4194150

[7] Huang Y , Xu W , Zhou R . NLRP3 inflammasome activation and cell death. Cell Mol Immunol. 2021;18:2114–2127. doi: 10.1038/s41423-021-00740-6 34321623 PMC8429580

[8] Paik S , Kim JK , Silwal P , Sasakawa C , Jo E‐K . An update on the regulatory mechanisms of NLRP3 inflammasome activation. Cell Mol Immunol. 2021;18:1141–1160. doi: 10.1038/s41423-021-00670-3 33850310 PMC8093260

[9] Swanson KV , Deng M , Ting JP‐Y . The NLRP3 inflammasome: molecular activation and regulation to therapeutics. Nat Rev Immunol. 2019;19:477–489. doi: 10.1038/s41577-019-0165-0 31036962 PMC7807242

[10] Mann DL . Innate immunity and the failing heart: the cytokine hypothesis revisited. Circ Res. 2015;116:1254–1268. doi: 10.1161/CIRCRESAHA.116.302317 25814686 PMC4380242

[11] Fuster JJ , MacLauchlan S , Zuriaga MA , Polackal MN , Ostriker AC , Chakraborty R , Wu C‐L , Sano S , Muralidharan S , Rius C , Vuong J , Jacob S , Muralidhar V , Robertson AAB , Cooper MA , Andrés V , Hirschi KK , Martin KA , Walsh K Clonal hematopoiesis associated with TET2 deficiency accelerates atherosclerosis development in mice. Science 2017;355:842–847, DOI: 10.1126/science.aag1381. 28104796 PMC5542057

[12] Sano S , Oshima K , Wang Y , MacLauchlan S , Katanasaka Y , Sano M , Zuriaga MA , Yoshiyama M , Goukassian D , Cooper MA , Fuster JJ , Walsh K Tet2‐mediated clonal hematopoiesis accelerates heart failure through a mechanism involving the IL‐1β/NLRP3 inflammasome. J Am Coll Cardiol 2018;71:875–886, DOI: 10.1016/j.jacc.2017.12.037. 29471939 PMC5828038

[13] Xiao H , Li H , Wang JJ , Zhang JS , Shen J , An XB , Zhang CC , Wu JM , Song Y , Wang XY , Yu HY , Deng XN , Li ZJ , Xu M , Lu ZZ , du J , Gao W , Zhang AH , Feng Y , Zhang YY IL‐18 cleavage triggers cardiac inflammation and fibrosis upon β‐adrenergic insult. Eur Heart J 2018;39:60–69, DOI: 10.1093/eurheartj/ehx261. 28549109

[14] Suetomi T , Willeford A , Brand CS , Cho Y , Ross RS , Miyamoto S , Brown JH . Inflammation and NLRP3 inflammasome activation initiated in response to pressure overload by Ca^2+^/calmodulin‐dependent protein kinase II δ signaling in cardiomyocytes are essential for adverse cardiac remodeling. Circulation. 2018;138:2530–2544. doi: 10.1161/CIRCULATIONAHA.118.034621 30571348 PMC6309790

[15] Higashikuni Y , Liu W , Numata G , Tanaka K , Fukuda D , Tanaka Y , Hirata Y , Imamura T , Takimoto E , Komuro I , Sata M NLRP3 inflammasome activation through heart–brain interaction initiates cardiac inflammation and hypertrophy during pressure overload. Circulation 2023;147:338–355, DOI: 10.1161/CIRCULATIONAHA.122.060860. 36440584

[16] Yao C , Veleva T , Scott L , Cao S , Li L , Chen G , Jeyabal P , Pan X , Alsina KM , Abu‐Taha I , et al. Enhanced cardiomyocyte NLRP3 inflammasome signaling promotes atrial fibrillation. Circulation. 2018;138:2227–2242. doi: 10.1161/CIRCULATIONAHA.118.035202 29802206 PMC6252285

[17] Shao D , Kolwicz SC , Wang P , Roe ND , Villet O , Nishi K , Hsu Y‐WA , Flint GV , Caudal A , Wang W , et al. Increasing fatty acid oxidation prevents high‐fat diet‐induced cardiomyopathy through regulating parkin‐mediated mitophagy. Circulation. 2020;142:983–997. doi: 10.1161/CIRCULATIONAHA.119.043319 32597196 PMC7484440

[18] Woo S‐H , Kyung D , Lee SH , Park KS , Kim M , Kim K , Kwon H‐J , Won Y‐S , Choi I , Park Y‐J , Go DM , Oh JS , Yoon WK , Paik SS , Kim JH , Kim YH , Choi JH , Kim DY TXNIP suppresses the osteochondrogenic switch of vascular smooth muscle cells in atherosclerosis. Circ Res 2023;132:52–71, DOI: 10.1161/CIRCRESAHA.122.321538. 36448450 PMC9829043

[19] Tschopp J , Schroder K . NLRP3 inflammasome activation: the convergence of multiple signalling pathways on ROS production? Nat Rev Immunol. 2010;10:210–215. doi: 10.1038/nri2725 20168318

[20] Abplanalp WT , Cremer S , John D , Hoffmann J , Schuhmacher B , Merten M , Rieger MA , Vasa‐Nicotera M , Zeiher AM , Dimmeler S . Clonal hematopoiesis‐driver DNMT3A mutations alter immune cells in heart failure. Circ Res. 2021;128:216–228. doi: 10.1161/CIRCRESAHA.120.317104 33155517

[21] Al‐Qazazi R , Lima PDA , Prisco SZ , Potus F , Dasgupta A , Chen K‐H , Tian L , Bentley RET , Mewburn J , Martin AY , et al. Macrophage‐NLRP3 activation promotes right ventricle failure in pulmonary arterial hypertension. Am J Respir Crit Care Med. 2022;206:608–624. doi: 10.1164/rccm.202110-2274OC 35699679 PMC9716901

[22] Pavillard LE , Cañadas‐Lozano D , Alcocer‐Gómez E , Marín‐Aguilar F , Pereira S , Robertson AAB , Muntané J , Ryffel B , Cooper MA , Quiles JL , Bullón P , Ruiz‐Cabello J , Cordero MD NLRP3‐inflammasome inhibition prevents high fat and high sugar diets‐induced heart damage through autophagy induction. Oncotarget 2017;8:99740–99756, DOI: 10.18632/oncotarget.20763. 29245937 PMC5725128

[23] Zeng C , Duan F , Hu J , Luo B , Huang B , Lou X , Sun X , Li H , Zhang X , Yin S , Tan H NLRP3 inflammasome‐mediated pyroptosis contributes to the pathogenesis of non‐ischemic dilated cardiomyopathy. Redox Biol 2020;34:101523, DOI: 10.1016/j.redox.2020.101523. 32273259 PMC7327979

[24] Heijman J , Muna AP , Veleva T , Molina CE , Sutanto H , Tekook M , Wang Q , Abu‐Taha IH , Gorka M , Künzel S , el‐Armouche A , Reichenspurner H , Kamler M , Nikolaev V , Ravens U , Li N , Nattel S , Wehrens XHT , Dobrev D Atrial myocyte NLRP3/CaMKII nexus forms a substrate for postoperative atrial fibrillation. Circ Res 2020;127:1036–1055, DOI: 10.1161/CIRCRESAHA.120.316710. 32762493 PMC7604886

[25] Enzan N , Matsushima S , Ikeda S , Okabe K , Ishikita A , Yamamoto T , Sada M , Miyake R , Tsutsui Y , Nishimura R , Toyohara T , Ikeda Y , Shojima Y , Miyamoto HD , Tadokoro T , Ikeda M , Abe K , Ide T , Kinugawa S , Tsutsui H ZBP1 protects against mtDNA‐induced myocardial inflammation in failing hearts. Circ Res 2023;132:1110–1126, DOI: 10.1161/CIRCRESAHA.122.322227. 36974722 PMC10144299

[26] Deng Y , Xie M , Li Q , Xu X , Ou W , Zhang Y , Xiao H , Yu H , Zheng Y , Liang Y , Jiang C , Chen G , du D , Zheng W , Wang S , Gong M , Chen Y , Tian R , Li T Targeting mitochondria‐inflammation circuit by β‐hydroxybutyrate mitigates HFpEF. Circ Res 2021;128:232–245, DOI: 10.1161/CIRCRESAHA.120.317933. 33176578

[27] Sandanger Ø , Ranheim T , Vinge LE , Bliksøen M , Alfsnes K , Finsen AV , Dahl CP , Askevold ET , Florholmen G , Christensen G , Fitzgerald KA , Lien E , Valen G , Espevik T , Aukrust P , Yndestad A The NLRP3 inflammasome is up‐regulated in cardiac fibroblasts and mediates myocardial ischaemia‐reperfusion injury. Cardiovasc Res 2013;99:164–174, DOI: 10.1093/cvr/cvt091. 23580606

[28] Sokolova M , Sjaastad I , Louwe MC , Alfsnes K , Aronsen JM , Zhang L , Haugstad SB , Bendiksen BA , Øgaard J , Bliksøen M , Lien E , Berge RK , Aukrust P , Ranheim T , Yndestad A NLRP3 inflammasome promotes myocardial remodeling during diet‐induced obesity. Front Immunol 2019;10:1621, DOI: 10.3389/fimmu.2019.01621. 31379826 PMC6648799

[29] Raggi F , Rossi C , Faita F , Distaso M , Kusmic C , Solini A . P2X7 receptor and heart function in a mouse model of systemic inflammation due to high fat diet. J Inflamm Res. 2022;15:2425–2439. doi: 10.2147/JIR.S356038 35444452 PMC9015053

[30] Li H , Guan Y , Liang B , Ding P , Hou X , Wei W , Ma Y . Therapeutic potential of MCC950, a specific inhibitor of NLRP3 inflammasome. Eur J Pharmacol. 2022;928:175091. doi: 10.1016/j.ejphar.2022.175091 35714692

[31] Wu D , Chen Y , Sun Y , Gao Q , Li H , Yang Z , Wang Y , Jiang X , Yu B . Target of MCC950 in inhibition of NLRP3 inflammasome activation: a literature review. Inflammation. 2020;43:17–23. doi: 10.1007/s10753-019-01098-8 31646445

[32] Coll RC , Hill JR , Day CJ , Zamoshnikova A , Boucher D , Massey NL , Chitty JL , Fraser JA , Jennings MP , Robertson AAB , Schroder K MCC950 directly targets the NLRP3 ATP‐hydrolysis motif for inflammasome inhibition. Nat Chem Biol 2019;15:556–559, DOI: 10.1038/s41589-019-0277-7. 31086327

[33] Coll RC , Robertson AAB , Chae JJ , Higgins SC , Muñoz‐Planillo R , Inserra MC , Vetter I , Dungan LS , Monks BG , Stutz A , Croker DE , Butler MS , Haneklaus M , Sutton CE , Núñez G , Latz E , Kastner DL , Mills KHG , Masters SL , Schroder K , Cooper MA , O'Neill LAJ A small‐molecule inhibitor of the NLRP3 inflammasome for the treatment of inflammatory diseases. Nat Med 2015;21:248–255, DOI: 10.1038/nm.3806. 25686105 PMC4392179

[34] Xing Y , Yao X , Li H , Xue G , Guo Q , Yang G , An L , Zhang Y , Meng G . Cutting edge: TRAF6 mediates TLR/IL‐1R signaling‐induced nontranscriptional priming of the NLRP3 inflammasome. J Immunol. 2017;199:1561–1566. doi: 10.4049/jimmunol.1700175 28739881

[35] Fang C , Wei X , Wei Y . Mitochondrial DNA in the regulation of innate immune responses. Protein Cell. 2016;7:11–16. doi: 10.1007/s13238-015-0222-9 26498951 PMC4707157

[36] Basnet R , Basnet TB , Basnet BB , Khadka S . Overview on thioredoxin‐interacting protein (TXNIP): a potential target for diabetes intervention. Curr Drug Targets. 2022;23:761–767. doi: 10.2174/1389450123666220303092324 35240955

[37] Gao C , Wang R , Li B , Guo Y , Yin T , Xia Y , Zhang F , Lian K , Liu Y , Wang H , Zhang L , Gao E , Yan W , Tao L TXNIP/Redd1 signalling and excessive autophagy: a novel mechanism of myocardial ischaemia/reperfusion injury in mice. Cardiovasc Res 2020;116:645–657, DOI: 10.1093/cvr/cvz152. 31241142

[38] Shi X , Han B , Zhang B , Chu Z , Zhang X , Lu Q , Han J . Schisandra chinensis polysaccharides prevent cardiac hypertrophy by dissociating thioredoxin‐interacting protein/thioredoxin‐1 complex and inhibiting oxidative stress. Biomed Pharmacother. 2021;139:111688. doi: 10.1016/j.biopha.2021.111688 34243612

[39] Yoshioka J , Imahashi K , Gabel SA , Chutkow WA , Burds AA , Gannon J , Schulze PC , MacGillivray C , London RE , Murphy E , Lee RT Targeted deletion of thioredoxin‐interacting protein regulates cardiac dysfunction in response to pressure overload. Circ Res 2007;101:1328–1338, DOI: 10.1161/CIRCRESAHA.106.160515. 17916779

[40] Li A , Zhang Y , Wang J , Zhang Y , Su W , Gao F , Jiao X . Txnip gene knockout ameliorated high‐fat diet‐induced cardiomyopathy via regulating mitochondria dynamics and fatty acid oxidation. J Cardiovasc Pharmacol. 2023;81:423–433. doi: 10.1097/FJC.0000000000001414 36888974 PMC10237349

[41] Wang M , Zhao M , Yu J , Xu Y , Zhang J , Liu J , Zheng Z , Ye J , Wang Z , Ye D , Feng Y , Xu S , Pan W , Wei C , Wan J MCC950, a selective NLRP3 inhibitor, attenuates adverse cardiac remodeling following heart failure through improving the cardiometabolic dysfunction in obese mice. Front Cardiovasc Med 2022;9:727474, DOI: 10.3389/fcvm.2022.727474. 35647084 PMC9133382

[42] Krishnan SM , Ling YH , Huuskes BM , Ferens DM , Saini N , Chan CT , Diep H , Kett MM , Samuel CS , Kemp‐Harper BK , Robertson AAB , Cooper MA , Peter K , Latz E , Mansell AS , Sobey CG , Drummond GR , Vinh A Pharmacological inhibition of the NLRP3 inflammasome reduces blood pressure, renal damage, and dysfunction in salt‐sensitive hypertension. Cardiovasc Res 2019;115:776–787, DOI: 10.1093/cvr/cvy252. 30357309 PMC6432065

[43] Lin Q , Li S , Jiang N , Jin H , Shao X , Zhu X , Wu J , Zhang M , Zhang Z , Shen J , Zhou W , Gu L , Lu R , Ni Z Inhibiting NLRP3 inflammasome attenuates apoptosis in contrast‐induced acute kidney injury through the upregulation of HIF1A and BNIP3‐mediated mitophagy. Autophagy 2021;17:2975–2990, DOI: 10.1080/15548627.2020.1848971. 33345685 PMC8525960

[44] Shi W , Chen J , Zhao N , Xing Y , Liu S , Chen M , Fang W , Zhang T , Li L , Zhang H , Zhang M , Zeng X , Chen S , Wang S , Xie S , Deng W Targeting heat shock protein 47 alleviated doxorubicin‐induced cardiotoxicity and remodeling in mice through suppression of the NLRP3 inflammasome. J Mol Cell Cardiol 2024;186:81–93, DOI: 10.1016/j.yjmcc.2023.11.007. 37995517

[45] Xie S , Zhang M , Shi W , Xing Y , Huang Y , Fang W‐X , Liu SQ , Chen MY , Zhang T , Chen S , Zeng X , Wang S , Deng W , Tang Q Long‐term activation of glucagon‐like peptide‐1 receptor by dulaglutide prevents diabetic heart failure and metabolic remodeling in type 2 diabetes. J Am Heart Assoc 2022;11:e026728, DOI: 10.1161/JAHA.122.026728. 36172969 PMC9673690

[46] Xie SY , Liu SQ , Zhang T , Shi WK , Xing Y , Fang WX , Zhang M , Chen MY , Xu SC , Fan MQ , Li LL , Zhang H , Zhao N , Zeng ZX , Chen S , Zeng XF , Deng W , Tang QZ USP28 serves as a key suppressor of mitochondrial morphofunctional defects and cardiac dysfunction in the diabetic heart. Circulation 2024;149:684–706, DOI: 10.1161/CIRCULATIONAHA.123.065603. 37994595

[47] Xie S , Chen M , Fang W , Liu S , Wu Q , Liu C , Xing Y , Shi W , Xu M , Zhang M , Chen S , Zeng X , Wang S , Deng W , Tang Q Diminished arachidonate 5‐lipoxygenase perturbs phase separation and transcriptional response of Runx2 to reverse pathological ventricular remodeling. EBioMedicine 2022;86:104359, DOI: 10.1016/j.ebiom.2022.104359. 36395739 PMC9672960

[48] Xie S , Xing Y , Shi W , Zhang M , Chen M , Fang W , Liu S , Zhang T , Zeng X , Chen S , Wang S , Deng W , Tang Q Cardiac fibroblast heat shock protein 47 aggravates cardiac fibrosis post myocardial ischemia‐reperfusion injury by encouraging ubiquitin specific peptidase 10 dependent Smad4 deubiquitination. Acta Pharm Sin B 2022;12:4138–4153, DOI: 10.1016/j.apsb.2022.07.022. 36386478 PMC9643299

[49] Xie S , Deng W , Chen J , Wu QQ , Li H , Wang J , Wei L , Liu C , Duan M , Cai Z , Xie Q , Hu T , Zeng X , Tang Q Andrographolide protects against adverse cardiac remodeling after myocardial infarction through enhancing Nrf2 signaling pathway. Int J Biol Sci 2020;16:12–26, DOI: 10.7150/ijbs.37269. 31892842 PMC6930369

