# Supplementary material for: Impeding Nucleotide‐Binding Oligomerization Domain‐Like Receptor 3 Inflammasome Ameliorates Cardiac Remodeling and Dysfunction in Obesity‐Associated Cardiomyopathy
Source: J Am Heart Assoc. 2024 Nov 27;13(23):e035234. doi: 10.1161/JAHA.124.035234 (PMC11681593; doi:10.1161/JAHA.124.035234)
Supplement: Supplementary file 1 — Data S1 Tables S1–S3 Figures S1–S7 References 44–49 [file JAH3-13-e035234-s001.pdf]

SUPPLEMENTAL MATERIAL

Data S1. Supplemental Methods

Animal studies and ethics

All animal experiments were performed following the Guide for the Care and Use of Laboratory Animals (NIH Publication, revised 2011) and were approved by the Animal Care and Use Committee of the Wuhan University People's Hospital (IvD number: WDRM 20210704A). C57/BL6N mice wild type (WT) aged 8-10 weeks were purchased from the Institute of Laboratory Animal Science (Beijing, China). NLRP3 knockout mice 5-6 weeks old were purchased from Jackson Laboratories[42]. Recombinant adeno-associated virus serotype 9 encoding TXNIP under cTnT promoter (rAAV9-cTnT-TXNIP) was injected in WT mice and NLRP3 knockout mice by tail vein. Two weeks later, the efficiency of rAAV9-mediated TXNIP gene overexpression was detected by western blotting. All mice were kept in a specific pathogen-free environment at a suitable temperature (20–25 °C) and humidity (50 ± 5%). The mice had free access to food and water. Mice were acclimatized for one week before the experiments. Mice were fed a high-fat diet (Xietong Shengwu Research Institute, D12492, 60 kcal% fat) for 24 weeks to induce obesity cardiomyopathy as described previously[17,43,44]. Control mice were fed a normal diet (ND) for 24 weeks (Table S1). MCC950 was intraperitoneally administered to obese mice after 12 weeks of HFD consumption at 10 mg/kg/d for 12 weeks to investigate the role of NLRP3 inflammasome in obese heart. Control mice received an equal volume of normal saline (NS) intraperitoneally at the same time. To investigate the role of mitochondrial ROS

in the NLRP3 inflammasome activation of the obese heart, mitochondrial ROS scavenger (MitoTEMPO) was injected into obese mice at 10 mg/kg/d for 12 weeks prior to aberrant NLRP3 inflammasome activation. Control mice received an equal volume of NS intraperitoneally at the same time. Mice were fasted overnight for 12 h (20:00 pm–8:00 am) before measuring fasting blood glucose (FBG). After 24 weeks of ND or HFD feeding, all mice were subjected to echocardiography to assess cardiac function and sacrificed for further experiments.

Adeno-associated virus and viral delivery protocol

To specifically overexpress TXNIP in the cardiomyocytes, recombinant adeno-associated virus 9 (AAV9) carrying the TXNIP gene under the cTnT promoter (rAAV9-cTnT-TXNIP) was injected intravenously via the tail vein in a single dose into the indicated mice. The NCBI accession number of the coding sequence used for the expressed transgene is NM 00100993. AAV9 without carrying USP28 gene (rAAV-null) was injected as a control vector. Specifically, 200μl NS mixed with ≥5×1011 vg rAAV9-cTnT-TXNIP recombinant was injected into indicated mice via tail vein, while the control mice were injected with an equal volume of NS mixed with rAAV9-null recombinant according to our previous studies[44,45]. Two weeks after adeno-associated virus injection, we assayed its overexpression efficiency. Western blot was used to detect Txnip expression levels in 8-week-old wild-type mice.

Echocardiography

To evaluate cardiac function, echocardiography was performed using Vevo® 3100 High-Resolution Preclinical Imaging System (FUJIFILM Visual Sonics) as we

previously described[46]. Briefly, inhalation anesthesia was induced in mice using 3% isoflurane. Afterwards, the isoflurane concentration was lowered to 1.0-1.5% to keep the heart rate of the mice at about 450 beats per minute. Cardiac contractile function was obtained from the short-axis M-mode at the midventricular level, where the papillary muscles could be visualized. In apical four-chamber views of the mice heart, we assessed cardiac diastolic function using pulsed-wave and tissue Doppler imaging at the level of the mitral valve. In this study, left ventricular (LV) fractional shortening and LV ejection fraction were utilized to evaluate cardiac systolic function. Diastolic function was assessed by early to late diastolic transmitral flow velocity (E/A ratio), transmitral flow (E wave), and E wave deceleration time. All parameters obtained by echocardiography were measured at least three times and the average values were calculated.

Histological analysis

Hearts were fixed with 10% neutral formalin buffer overnight. After dehydration and paraffin embedding, the heart was cut transversely into 4.0 μm thick slices. As described previously[45,47], Hematoxylin and eosin (HE) staining was used to assess the degree of myocardial hypertrophy. Cardiac fibrosis was determined by Masson's trichrome staining. Cardiac hypertrophy was specifically assessed by Wheat germ agglutinin staining (WGA), as shown by the cross-sectional area of cardiomyocytes. These staining results were analyzed by image J software, and over 50 fields per group were analyzed. Tissue immunofluorescence assays was used to detect NLRP3 and TXNIP expression levels, co-localization of NLRP3 and TXNIP. Sections were heated

by microwave heating in citrate buffer for antigen repair and 10% goat serum was used to reduce nonspecific background staining. Cardiac sections were incubated with anti-NLRP3 and anti-TXNIP antibodies (1: 100; diluted in PBS) overnight at 4°C, followed by incubation with secondary antibody (1: 200; diluted in PBS) for 1 hour. Finally, DAPI was used to display nuclei. Images were captured using a fluorescence microscope (Apotome, Zeiss, Oberkochen, Germany) and quantified using ImageJ software. Immunohistochemical staining was utilized to detect the expression of IL-1β and NF-κB in indicated heart. After dewaxing, hydration, and antigen repair by microwave heating in citrate buffer, sections were treated with 3% hydrogen peroxide to inhibit endogenous peroxidase, followed by occlusion with 8% goat serum for 30 minutes at room temperature. Sections were then incubated with anti- IL-1β and anti-NF-κB antibodies (1: 100; diluted in PBS) at 4°C overnight, followed by further processing with anti-rabbit/mouse EnVision™+/HRP reagent (Gene Technology, China) for 15 minutes. Finally, samples were visualized using diaminobenzidine and nuclei were stained with hematoxylin. Images were acquired using an Olympus BX41 microscope (Olympus, Milan, Italy) and analyzed by two laboratory staff who were not aware of the experimental mice groups. The staining results were analyzed by image J software, and over 50 fields per group were analyzed. For tissue Oil Red O staining, we used an oil red O kit (G1262, Solarbio) to assess lipid content in myocardial tissues as described in our previous study[43,44].

Neonatal rat cardiomyocytes isolation and culture

Neonatal rat ventricular myocytes (NRVMs) were isolated as previously described[48,49],

and their purity were identified by immunofluorescence staining of F-actin. Isolated NRVMs were cultured in DMEM containing 1.0g/L D-glucose supplemented with 10% FBS, 100 U/mL penicillin and 0.1 mg/mL streptomycin at 37°C in a 5% CO2 incubator for subsequent experiments. After being starved for 12 hours, cells were incubated in serum-free medium supplemented with 500mM bovine serum albumin plus 400 μM palmitic acid (BSA+PA) for 24 hours to mimic obesity cardiomyopathy in vitro. Control cells were stimulated with 500mM bovine serum albumin without palmitic acid. Palmitic acid was dissolved in ethanol to form a 400 mmol/L stock solution and stored at -20°C. To stimulate NRVCM with palmitic acid at physiological concentrations of albumin, 500 mmol/L stock solution of palmitic acid was added to serum-free medium containing 500mM bovine serum albumin and diluted to a concentration of 400 μM. NRVMs were treated with MCC950 for 24 hours to inhibit NLRP3 inflammasome activation. MitoTEMPO was applied to the experiment to investigate the role of mitochondrial reactive oxygen species on NLRP3 inflammasome activation in BSA+PA-treated NRVMs.

siRNA transfection in NRVMs

To knockdown endogenous TXNIP in vitro, NRVMs were preincubated with small interfering RNA (siTXNIP, 50 nmol/) against TXNIP with Lipo-6000 transfection reagent (Beyotime, China) for 48 hours before BSA+PA stimulation. The siTXNIP sequence used in this experiment was AACATCCTTTAAAGGAAAATATG. Scramble RNA (siRNA) was used as the negative control. Cell protein was extracted and the efficiency of siRNA transfection was determined by western blotting. Cell viability was

measured using the CCK-8 Assay Kit (C0038, Beo Tianmei, China) according to the manufacturer's instructions.

Cell immunofluorescence assay

In the study, immunofluorescence assays were used to detect NLRP3 and ASC protein expression levels, NLRP3 and NF-κB cellular localization, and co-localization of NLRP3 and TXNIP in NRVMs. Briefly, after 15 minutes of fixation with 4% paraformaldehyde and permeabilization in 0.2% Triton X-100 for 10 min at room temperature, NRVMs were treated with 8% goat serum for 1 hour to reduce nonspecific background staining. Cells were then incubated with primary antibodies (1: 100; diluted in PBS) overnight at 4°C followed by incubation with secondary antibody (1: 200; diluted in PBS) for 1 hour at 37°C. Finally, dropwise addition of DAPI allowed visualization of nuclei. Images were captured using a fluorescence microscope and quantified using ImageJ software.

Protein extraction and immunoblotting

Heart was quickly harvested and rinsed in saline. Left ventricular myocardial tissue was rapidly dropped into liquid nitrogen and then stored at -80°C. Myocardial tissue was ground into a homogenate and then lysed in 1x RIPA lysis buffer (Invitrogen, Carlsbad, CA, USA) containing phosphatase and protease inhibitors. NRVMs were directly lysed on ice with 1 x RIPA containing protease and phosphatase inhibitors. Importantly, we utilized Nuclear and Cytoplasmic Protein Extraction Kits (Beyotime, P0027) to extract nuclear and cytoplasmic proteins from cells for subsequent study. Protein concentrations was determined by Bicinchoninic Acid protein quantification kit

(Servicebio, G2026). Protein was separated by 10% or 12% SDS-PAGE and then transferred to PVDF membranes. After an hour of closure with 5% skim milk powder, the membrane was incubated with specific primary antibodies overnight at 4°C. After incubation with the secondary antibodies at room temperature for one hour, the membrane was visualized using ECL Substrate Kit (Biosharp, BL520B). Protein bands were analyzed by image J software. Nuclear proteins and cytoplasmic proteins were normalized to Lamin B1 and GAPDH respectively.

Measurement of serum triglyceride levels

The serum triglyceride was measured using mouse enzyme-linked immunosorbent assay kits (Nanjing Jiancheng Bioengineering institute, A110-1-1). The kit is based on the measurement of glycerol produced by TG hydrolysis. Lipase was used to break down triglycerides to produce glycerol and fatty acids. Triglyceride levels were subsequently determined by measuring glycerol content.

Measurement of IL-1β and IL-18 in the heart

Mouse IL-1 beta ELISA Kit (Servicebio, GEM0002) and Mouse IL-18 ELISA Kit (Servicebio, GEM0010) were used to assess IL-1β and IL-18 in mice hearts respectively. UV spectrophotometer with Gen5 software was used for data collection and processing. Values measured by ELISA are normalized to the weight of the myocardium. All ELISA kits were operated according to the manufacturer's instructions.

DCFH-DA and MitoSOX Staining

To detect total ROS production, NRVMs were incubated with 10 μM DCFH-DA fluorescent probe for 15 min at 37 °C (ROS Assay Kit, Biyotime). MitoSOX Red

probes (10 μM at 37°C for 30 min, Invitrogen, M36008) was used to detect mitochondrial ROS production in BSA+PA-treated NRVMs. Cells were then washed with PBS and investigated with a fluorescent microscope.

Co-Immunoprecipitation (IP) assay

Fresh heart tissue was grinded at 60 Hz for 120 seconds at 4°C using a grinder. Heart tissue homogenate or cells were lysed on ice for 15 minutes after the addition of immunoprecipitation buffer (Servicebio, G2038). The supernatant containing total protein was obtained after centrifugation. Bicinchoninic Acid protein quantification kit (Servicebio, G2026) was used to determined protein concentrations. In the study, sample lysates containing 1 mg protein were used to perform immunoprecipitation. Protein lysates were incubated with anti-TXNIP antibody and rotated at 4°C for 5 hours. Subsequently, 20 μL protein A/G-agarose beads were added into the lysates and the mixture was pulled down after 2 hours of incubation. After being washed with the immunoprecipitation buffer, protein mixture obtained was boiled at 95°C for 5 minutes with an equal volume of 2×SDS loading buffer (Servicebio, G2076).

Cell Oil Red O staining

Saturated Oil Red O dye solution (Servicebio, G1015) was utilized to detect lipid droplets in cells as we illustrated previously[44]. The saturated Oil Red O dye solution was mixed with ddH2O in the ratio of 3:2, and the mixture was incubated in a water bath at 65°C for 30 minutes and then filtered to obtain an oil-red colorant. and then filtered to obtain the Oil Red O working solution. Cells were washed with PBS, and fixed with 60% isopropanol. Oil Red O working solution was added to the cells and

incubated for 30 min at room temperature. Finally, the nuclei were stained with hematoxylin staining solution.

Statistical analysis

All results are presented as the mean ± SEM. All results were tested for normality with the Shapiro-Wilk normality test, and all data exhibited a normal distribution in the study. Two-group comparisons were performed by unpaired Student's t-test, and data were analyzed by 2-way ANOVA and the Tukey post-test for comparison of multiple groups. Furthermore, one-way ANOVA was used for the time-course experiments. All statistical analyses were performed using GraphPad Prism 8.0 software

Table S1. Ingredient information for high-fat diet vs. normal diet.

	**Normal Diet (3.85 Kcal/g)**	**High-Fat Diet (5.24 Kcal/g)**
**Nutrition information**	% Kcal from	% Kcal from
Protein	20	20
Carbohydrate	70	20
Fat	10	60
Total	100	100
**Ingredient**	g/Kg	g/Kg
Casein, 30 Mesh	200	200
L-Cystine	3	3
Corn Starch	315	0
Maltodextrin 10	35	125
Sucrose	350	68.6
Cellulose, BW200	50	50
Soybean oil	25	25
Lard	20	245
Mineral Mix S10026	10	10
DiCalcium Phosphate	13	13
Calcium Carbonate	5.5	5.5
Potassium Citrate	16.5	16.5
Vitamin Mix V10001	10	10
Choline Bitartrate	2	2
FD&C Blue Dye #1	0.05	0.05
Total	1055.55	773.85

Table S2. Antibodies used in this study

Antibody	Customer	Product number	Application
NLRP3	Abcam	ab4207	WB, IF
TXNIP	Abcam	ab210826	WB, IF
TRX	Abcam	ab109385	WB
Collagen I	Abcam	ab138492	WB
Caspase-1	Santa Cruz	sc-56036	WB
Lamin B1	Proteintech	12987-1-AP	WB
BNP	ABclonal	A23996	WB
ASC	ABclonal	A1170	WB, IF
IL-1β	ABclonal	A1112	WB, IHC
IL-18	ABclonal	A1115	WB
Phospho-NFκB (Ser536)	CST	3033S	WB
CTGF	CST	86641S	WB
NFκB P65	CST	8242S	WB, IF, IHC
GAPDH	CST	2118S	WB

Table S3. Primers used for the RT-qPCR assays in this study

Primers for mouse RT-qPCR		
Gene	Forward primers (5'- 3')	Reverse primers (5'- 3')
Nlrp3	ATTACCCGCCCGAGAAAGG	CATGAGTGTGGCTAGATCCAAG
ASC	GACAGTGCAACTGCGAGAAG	CGACTCCAGATAGTAGCTGACAA
Caspase1	ACAAGGCACGGGACCTATG	TCCCAGTCAGTCCTGGAAATG
Il-1β	GAAATGCCACCTTTTGACAGTG	TGGATGCTCTCATCAGGACAG
IL-18	GTGAACCCCAGACCAGACTG	CCTGGAACACGTTTCTGAAAGA
Txnip	GGCCGGACGGGTAATAGTG	AGCGCAAGTAGTCCAAAGTCT
CD36	ATGGGCTGTGATCGGAACTG	TTTGCCACGTCATCTGGGTTT
Cpt1b	TCTTCTTCCGACAAACCCTGA	GAGACGGACACAGATAGCCC
Acadl	TTTCCTCGGAGCATGACATTTT	GCCAGCTTTTTCCCAGACCT
Acadvl	ACTACTGTGCTTCAGGGACAA	GCAAAGGACTTCGATTCTGCC
ANP	GTGCGGTGTCCAACACAGAT	TCCAATCCTGTCAATCCTACCC
BNP	GAGGTCACTCCTATCCTCTGG	GCCATTTCCTCCGACTTTTCTC
Myh7	CCTGCGGAAGTCTGAGAAGG	CTCGGGACACGATCTTGGC
Col1a1	GCTCCTCTTAGGGGCCACT	ATTGGGGACCCTTAGGCCAT
CTGF	GGCCTCTTCTGCGATTTCG	GCAGCTTGACCCTTCTCGG

Primers for Rat RT-qPCR		
Gene	Forward primers (5'- 3')	Reverse primers (5'- 3')
Nlrp3	ACCAGCCAGAGTGGAATGATG	ACAAATCGAGATGCGGGAGAG
ASC	GGCCTAAGCAGATGACTTCCT	TCAACTTCTGTGACCCTGGC
Caspase-1	AACACCCACTCGTACACGTC	TCCTTGTTTCTCTCCACGGC
IL-1β	TTGAGTCTGCACAGTTCCCC	GTCCTGGGGAAGGCATTAGG
IL-18	GCAACCCATTCCTCTGTGGT	ATAGGGTCACAGCCAGTCCT
CD36	CCGCTGTGGAAATGGTAGCTT	TGCCACAGCCAGATTGAGAA
Cpt1b	TATGGCTAACCGAAAGGGGG	GAGCCATACCTTGTCGGGAG
Acadl	TGGGAATGAAAGCCCAGGAC	GCAGCTGTCCACAAAAGCTC
Acadvl	GGCTTTGGAGATGCAGTCGG	GGCGTTTTGGCAAAGACAGT
BNP	CTGAGAGGCTAGTGCACGC	AGAATCATCTGGGGCAGCAC

Figure S1. NLRP3 inflammasome is activated in obesity cardiomyopathy. Related to figure 1.

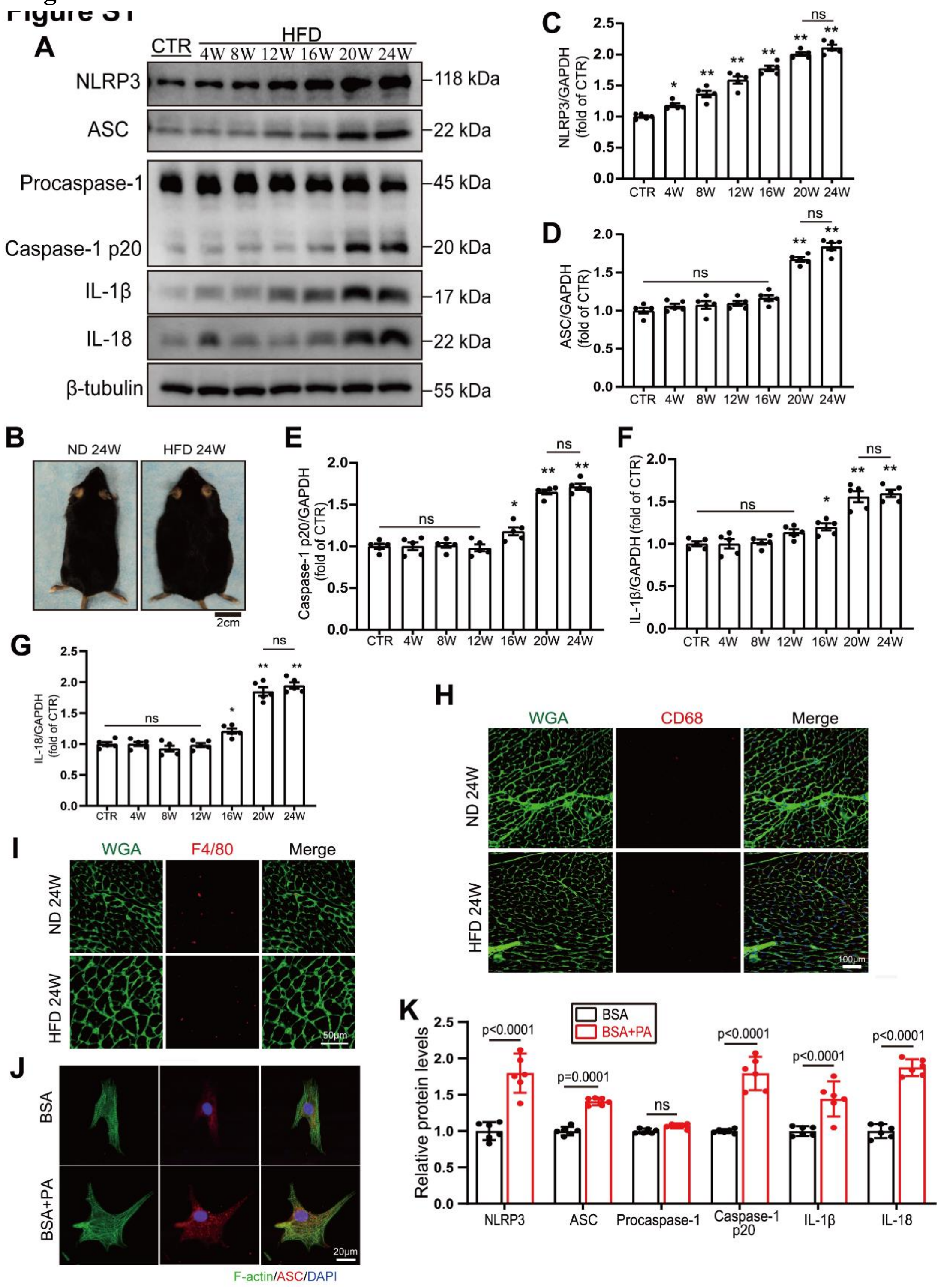

A-I. WT mice were subjected to ND or HFD feeding for different durations.

(A). Representative immunoblots of NLRP3, ASC, Caspase-1, active caspase-1 p20, IL-1β, and IL-18 in the whole-cell lysate of the heart at different time points following HFD consumption in indicated mice.

(B). Representative picture of mice after ND or HFD feeding for 24 weeks. Scale bar indicates 2cm.

(C-G). Quantitation of immunoblots of NLRP3, ASC, Caspase-1, active caspase-1 p20, IL-1β, and IL-18 in indicated heart of **Figure S1A** (n=6 per group).
(H). Representative images of wheat germ agglutinin staining (Green) and immunofluorescence staining of CD68 (Red) in the indicated heart.
(I). Representative images of wheat germ agglutinin staining (Green) and immunofluorescence staining of F4/80 (Red).
(J). Representative images of immunofluorescence staining of ASC (Red) and F-actin (Green) in NRVMs treated with LG or HG+PA for 24h.
(K). Quantitation of immunoblots of NLRP3, ASC, Procapase-1, cleaved Caspase-1 p20, IL-1β, and IL-18 protein of **Figure 1G** (n=6 independent experiments)**.**

Figure S2. NLRP3 deletion ameliorates obesity-induced cardiomyopathy. Related to figure 2.

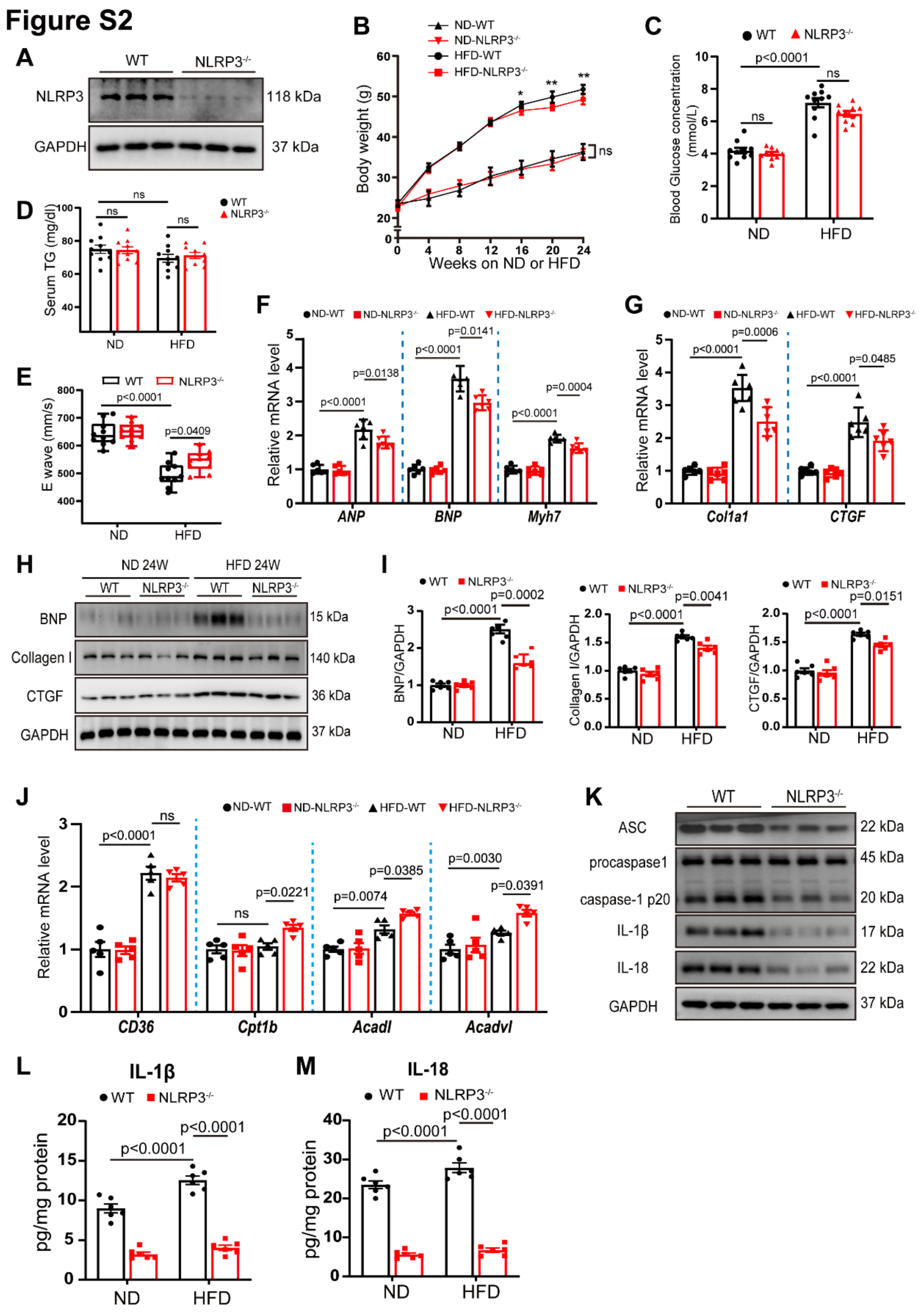

A. Verification of NLPR3 deletion in heart by immunoblot analysis of tissue lysates.
B-L. Wild-type and NLRP3$^{-/-}$ mice were subjected to ND or HFD feeding for 24 weeks.

B. Body weight from indicated mice after 4, 8, 12, 16, 20 and 24 weeks of HFD feeding.
C-D. The fasting blood glucose (FBG) **(C)** and serum triglyceride (TG) **(D)** from indicated mice after 24 weeks of HFD feeding (n=10 per group).
E. E wave was evaluated by transmitral flow Doppler echocardiography in indicated mice (n=10 per group).
F-G. qRT-PCR measurements of indicated genes involved in cardiac hypertrophy such as ANP, BNP, and β-MHC and cardiac fibrosis such as Collagen I and CTGF in the heart of indicated mice (n=6 per group).
H-I. Representative immunoblots of BNP, Collagen I and CTGF from heart tissues **(H)** and statistical analyses of densitometric measurements of BNP, Collagen I and CTGF **(I)** are shown (n=6 per group).
J. qRT-PCR detection of indicated genes related to fatty acid transport genes Cd36 and fatty acid oxidation genes Cpt1b, Acadl, and Acadvl in hearts from indicated mice (n=6 per group).
K. Representative immunoblots of ASC, Caspase-1, active caspase-1 p20, IL-1β, and IL-18 in the whole-cell lysate of the heart in indicated mice.
L-M. Protein of IL-1β (J) and IL-18 (K) in the indicated heart. IL-1β and IL-18 protein were detected by ELISA. Values were normalized to total protein level (n=6 per group).

Figure S3. MCC950 ameliorates obesity cardiomyopathy. Related to figure 3.

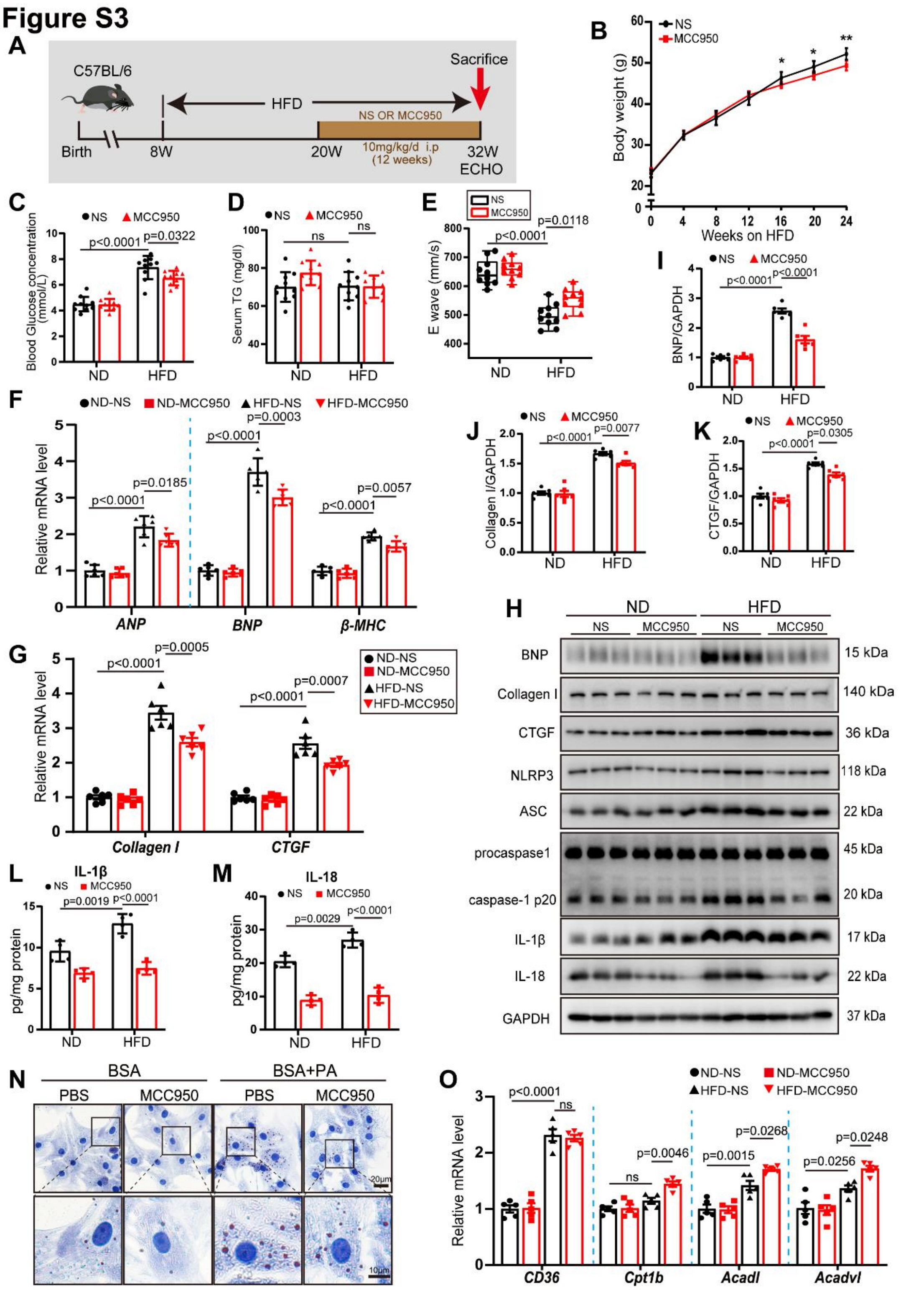

A. Experimental schematic diagram showing the process of HFD-induced obesity cardiomyopathy. After 12 weeks of HFD feeding, NLRP3 inflammasome inhibitor MCC950 was injected into obese mice at a dose of 10 mg/kg/d for 12 weeks.

B-O. Wild-type mice received NS or MCC950 were subjected to ND or HFD feeding for 24 weeks.

B. Body weight from indicated mice after 4, 8, 12, 16, 20 and 24 weeks of HFD feeding.
C-D. The fasting blood glucose (FBG) **(C)** and serum triglyceride (TG) **(D)** from indicated mice after 24 weeks of HFD feeding (n=10 per group).
E. E wave was evaluated by transmitral flow Doppler echocardiography in indicated mice (n=10 per group).
F-G. qRT-PCR measurements of indicated genes involved in cardiac hypertrophy such as ANP, BNP, and β-MHC and cardiac fibrosis such as Collagen I and CTGF in the heart of indicated mice (n=6 per group).
H-K. Representative immunoblots and quantification of BNP, Collagen I, CTGF, NLRP3 inflammasome components and proinflammatory cytokines in heart tissues from indicated mice (n=6 per group).
L-M. Protein of IL-1β (J) and IL-18 (K) in the indicated heart. IL-1β and IL-18 protein were detected by ELISA. Values were normalized to total protein level (n=6 per group).
N. Oil Red O (ORO) staining of NRVMs from indicated groups. Red indicates lipid droplets, blue indicates nuclei. NRVMs treated with PBS or MCC950 were incubated with BSA or BSA+PA medium for 24 hours.
O. qRT-PCR detection of indicated genes related to fatty acid transport genes Cd36 and fatty acid oxidation genes Cpt1b, Acadl, and Acadvl in hearts from indicated mice (n=6 independent experiments).

Figure S4. MCC950 inhibits NF-κB activation-mediated priming of NLRP3 inflammasome. Related to figure 4.

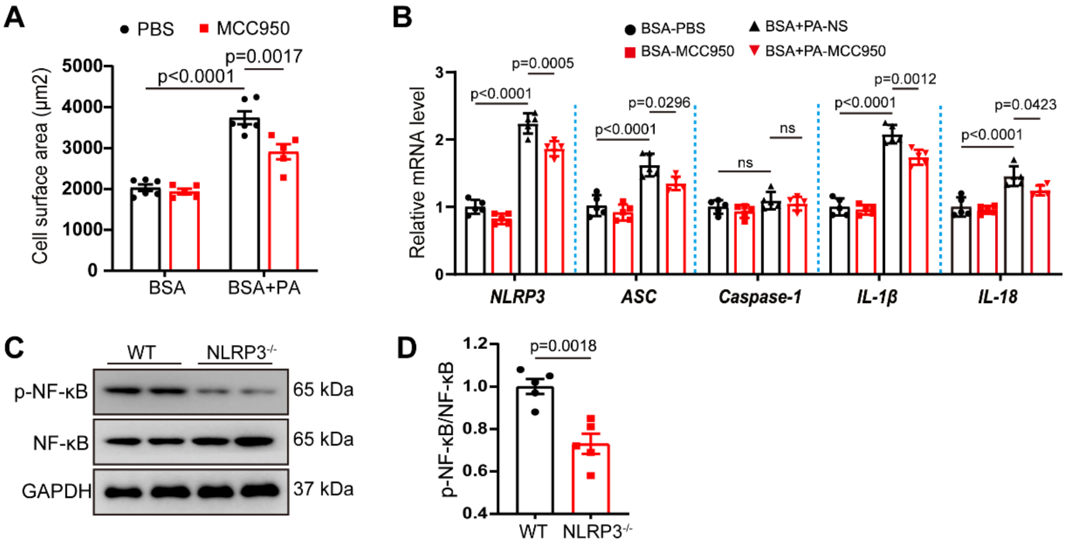

A-B. NRVMs treated with NS or MCC950 were incubated with BSA or BSA+PA medium for 24 hours.

A. Cell surface area of NRVMs (n=6 independent experiments)**.**

B. qRT-PCR detection of indicated genes related to NLRP3 inflammasome components and the proinflammatory cytokine IL-1β and IL-18 in NRVMs (n=6 independent experiments).

C-D. Representative images of immunoblot and quantitative analysis of NF-κB in heart tissues from WT and NLRP3$^{-/-}$ mice subjected to HFD for 24 weeks (n=5 per group).

Figure S5. NLRP3 deletion abrogates TXNIP overexpression-induced exacerbation of heart failure in obese hearts. Related to figure 5.

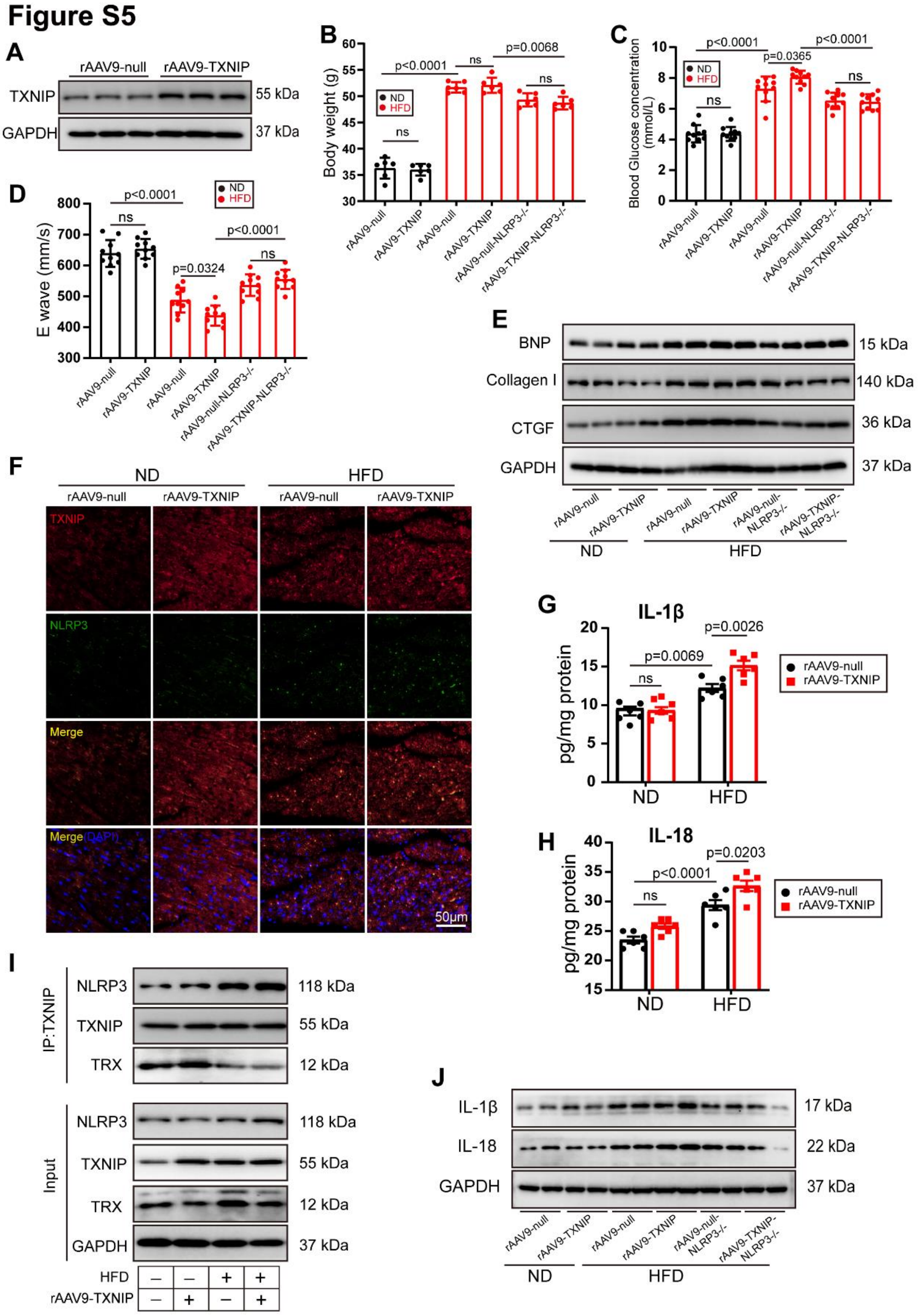

A. Verification of overexpression of TXNIP by immunoblot analysis of cell lysates from hearts of 8 week-old wild-type mice.

B-J. Wild-type and NLRP3$^{-/-}$ mice with or without TXNIP overexpression were subjected to ND or HFD feeding for 24 weeks.

B-C. Body weight from indicated mice after 24 weeks of ND or HFD feeding (n=6 per group).
C. The fasting blood glucose (FBG) from indicated mice after 24 weeks of ND or HFD feeding (n=10 per group).
D. E wave was evaluated by transmitral flow Doppler echocardiography in indicated mice (n=10 per group).
E. Representative immunoblots of BNP, Collagen I and CTGF in heart tissues from indicated mice.
F. Triple immunofluorescence (IF) staining for TXNIP (Red), NLRP3 (Green), and nuclei (DAPI, Blue) was performed in heart of indicated mice.
G-H. Protein of IL-1β (J) and IL-18 (K) in the indicated heart. IL-1β and IL-18 protein were detected by ELISA. Values were normalized to total protein level (n=6 per group).
I. Tissue lysate in heart of indicated mice were immunoprecipitated with TXNIP antibody, and immunoblot assays were performed using NLRP3, TXNIP, and TRX antibodies.
J. Representative immunoblots of ASC, Caspase-1, active caspase-1 p20, IL-1β, and IL-18 in the whole-cell lysate of the heart in indicated mice.

Figure S6. Inhibition of mitochondrial ROS ameliorates HG+PA-induced activation of TXNIP/NLRP3 inflammasome and impaired lipid metabolism. Related to figure 6.

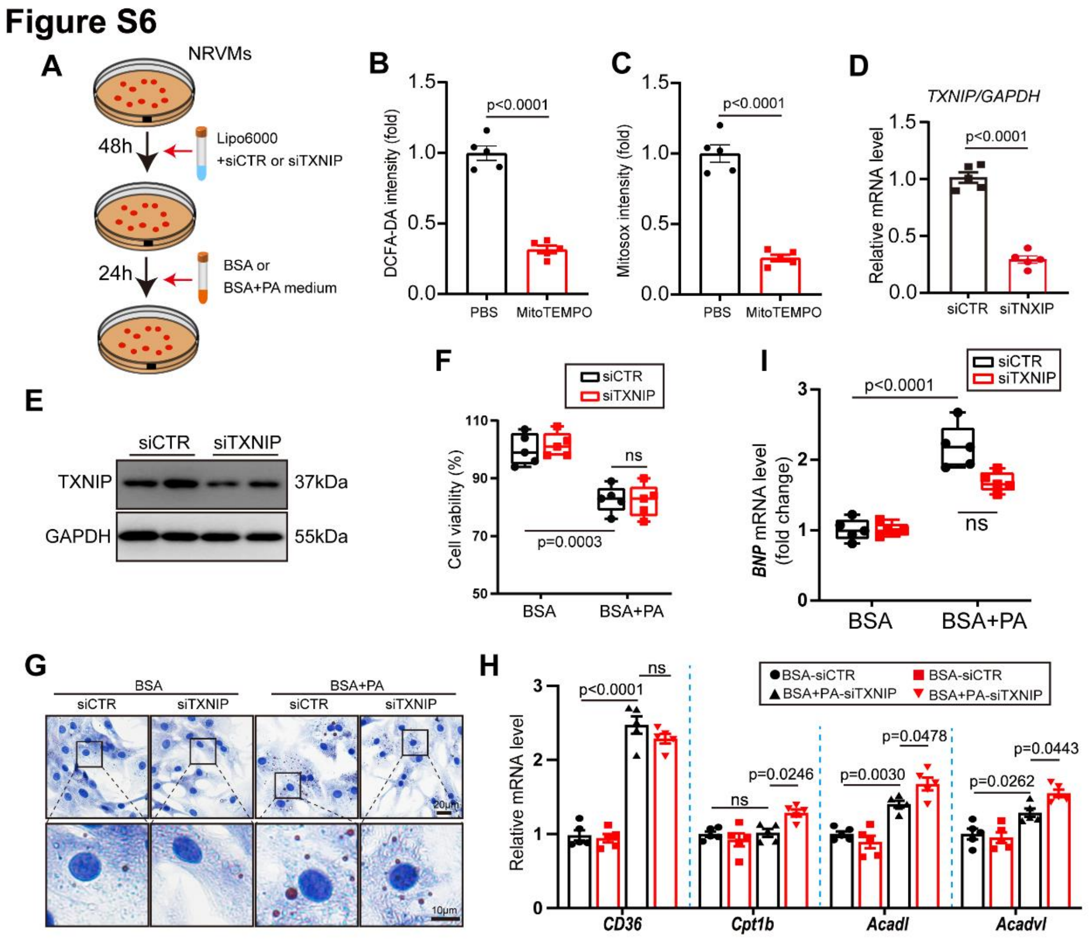

A. Schematic diagram showing the procedure of NRVMs.

B-C. Mean DCFH and Mitosox fluorescence intensity in NRVMs (n=5 independent experiments) .

D-E. Verification of knockdown of TXNIP in NRVMs by immunoblotting and RT-PCR (n=6 independent experiments).

F. Cell viability in indicated NRVMs was detected by CCK-8 assay (n=6 independent experiments).

G. Oil Red O (ORO) staining of NRVMs from indicated groups.

H. qRT-PCR detection of indicated genes related to fatty acid transport and oxidation genes (n=6 independent experiments).

I. Expression level of hypertrophic marker gene BNP was determined by real-time PCR, and normalized to that of GAPDH (n=6 independent experiments).

Figure S7. MitoTEMPO treatment alleviates NLRP3 inflammasome activation and rescues obesity-induced cardiomyopathy in vivo. Related to figure 7.

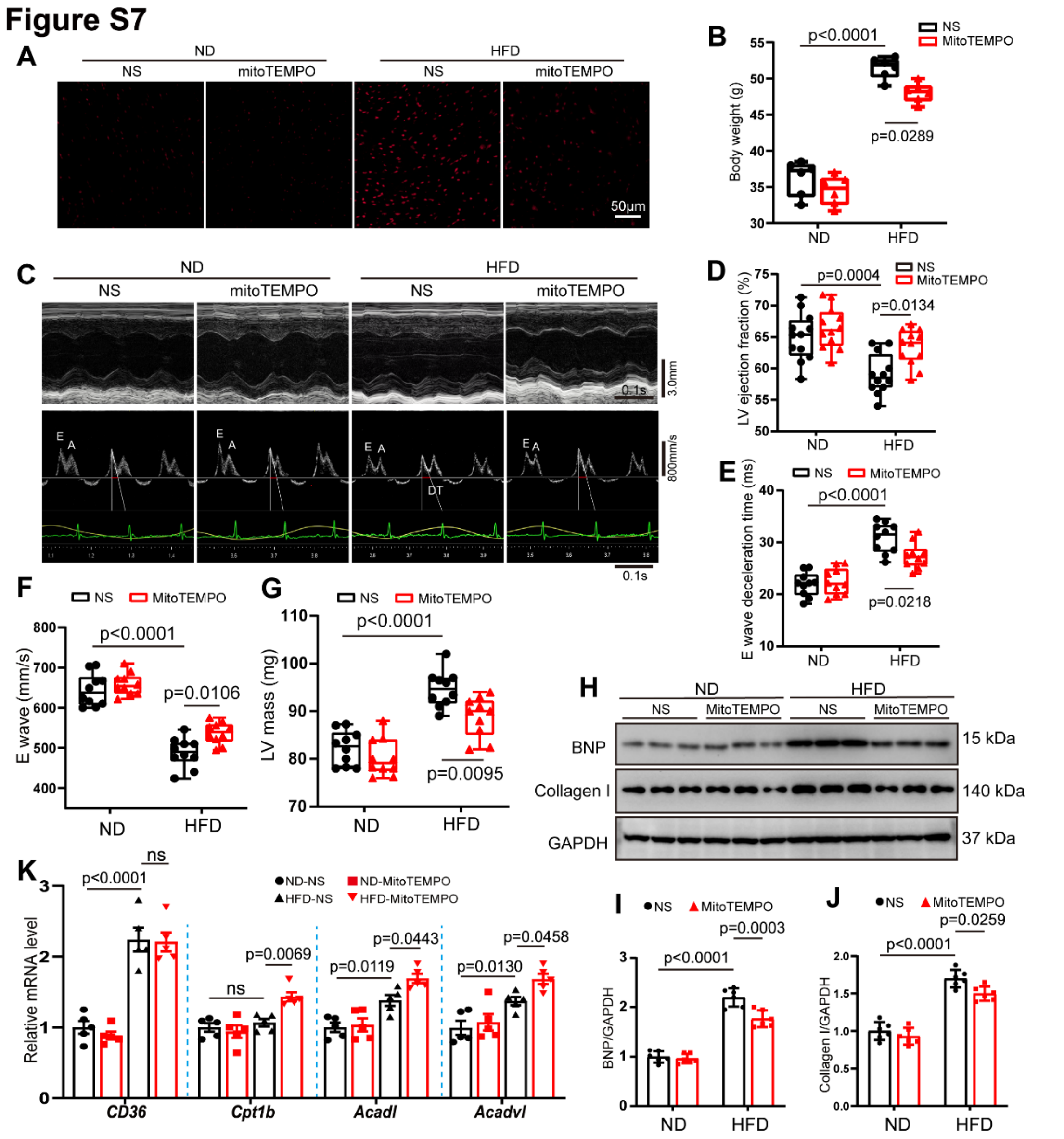

A. Representative dihydroethidium (DHE) staining and statistical analyses of ROS in the myocardium in indicated mice.

B. Body weight from indicated mice after 24 weeks of ND or HFD feeding (n=6 per group).

C. Representative images of M-model echocardiography (upper) and transmitral flow obtained by Doppler echocardiography (lower). E wave deceleration time (DT) is indicated by red bars.

D-G. Left ventricular (LV) ejection fraction **(D)**, E/A **(E)**, E wave **(F)** and LV mass **(G)** were measured by echocardiography in indicated mice (n=10 per group).

H-I. Representative immunoblots and quantification of BNP, Collagen I in heart tissues from indicated mice (n=6 per group).

J. qRT-PCR detection of indicated genes related to fatty acid transport and oxidation genes (n=6 per group).
